# Antibodies to synthetic citrullinated peptide epitope correlate with disease activity and flares in rheumatoid arthritis

**DOI:** 10.1371/journal.pone.0232010

**Published:** 2020-04-23

**Authors:** Sangita Khatri, Jonas Hansen, Kira Astakhova

**Affiliations:** 1 Department of Chemistry, Technical University of Denmark, Kgs Lyngby, Denmark; 2 Institute of Molecular Medicine, Sechenov First Moscow State Medical University, Moscow, Russia; Duke University School of Medicine, UNITED STATES

## Abstract

Rheumatoid arthritis (RA), caused by the abnormal recognition of human joint cells by autoimmune antibodies, remains the world’s most prevalent autoimmune disease, with over five million people affected and as much as 4% of the population at risk of RA. To prevent rapid disease development, hormonal and anti-inflammatory therapies require fast and reliable RA diagnosis. However, difficulty in detecting early specific biomarkers for RA means that it is unclear when treatment needs to begin. Here, we combined synthesis of citrullinated peptide epitopes with molecular diagnostics to verify a new specific biomarker for early RA diagnosis and flare prediction. A fibrinogen-derived 21-amino-acid-long citrullinated peptide showed high reactivity toward autoantibodies in RA samples. Additionally, the level of antibodies to this epitope was elevated prior to flares. In contrast, other citrullinated protein variants had lower reactivity and poorer sensitivity to disease activity. In conclusion, fibrinogen-derived epitope E2 subjected to citrullination facilitated a reliable RA diagnosis with a strong correlation to disease activity. This is of a high value for the diagnosis and management of RA patients who respond poorly to treatment.

## Introduction

Rheumatoid arthritis (RA) is one of the most common systemic inflammatory diseases; it is diagnosed in over 1% of the population [1]. RA diagnosis relies on clinical criteria and physical examination, including laboratory and radiographic results [2]. Hence, the RA diagnosis is typically established during its last stage.

Early RA diagnosis is highly desired to reach the optimal therapeutic window. Anti-citrullinated protein antibodies (ACPAs) are useful serological biomarker for RA diagnosis in over 50% subjects [3]. ACPA assays developed in the past decade have similar sensitivity but higher specificity than rheumatoid factor (RF), with positive predictive value for ACPA assay and specificity over 90%, versus only 60% for RF [2,4]. Furthermore, the presence of ACPA before the onset of RA symptoms can greatly advance understanding of disease pathogenesis. This knowledge may represent a milestone for early RA diagnosis and effective disease management [5].

Flares in RA are rather uncommon in case the current management scheme (i.e. treat to target) works well for a patient [6]. “Poor responders” is a group of RA patients that fails on established treatment schemes and is prompt to disease flares. It has been reported that failure on methotrexate (MTX) and on a following disease modifying anti-rheumatic drugs (DMARD)/biologics treatment leads to an increased flare rate [6]. According to 2018 report, flares are common on a 12-month treatment span for the RA patients with low disease activity, which is not dependent on their biomarker status [7]. Importantly, patients who flared had significantly worse outcomes after a 12-month long treatment [7].

**Fig 1:**
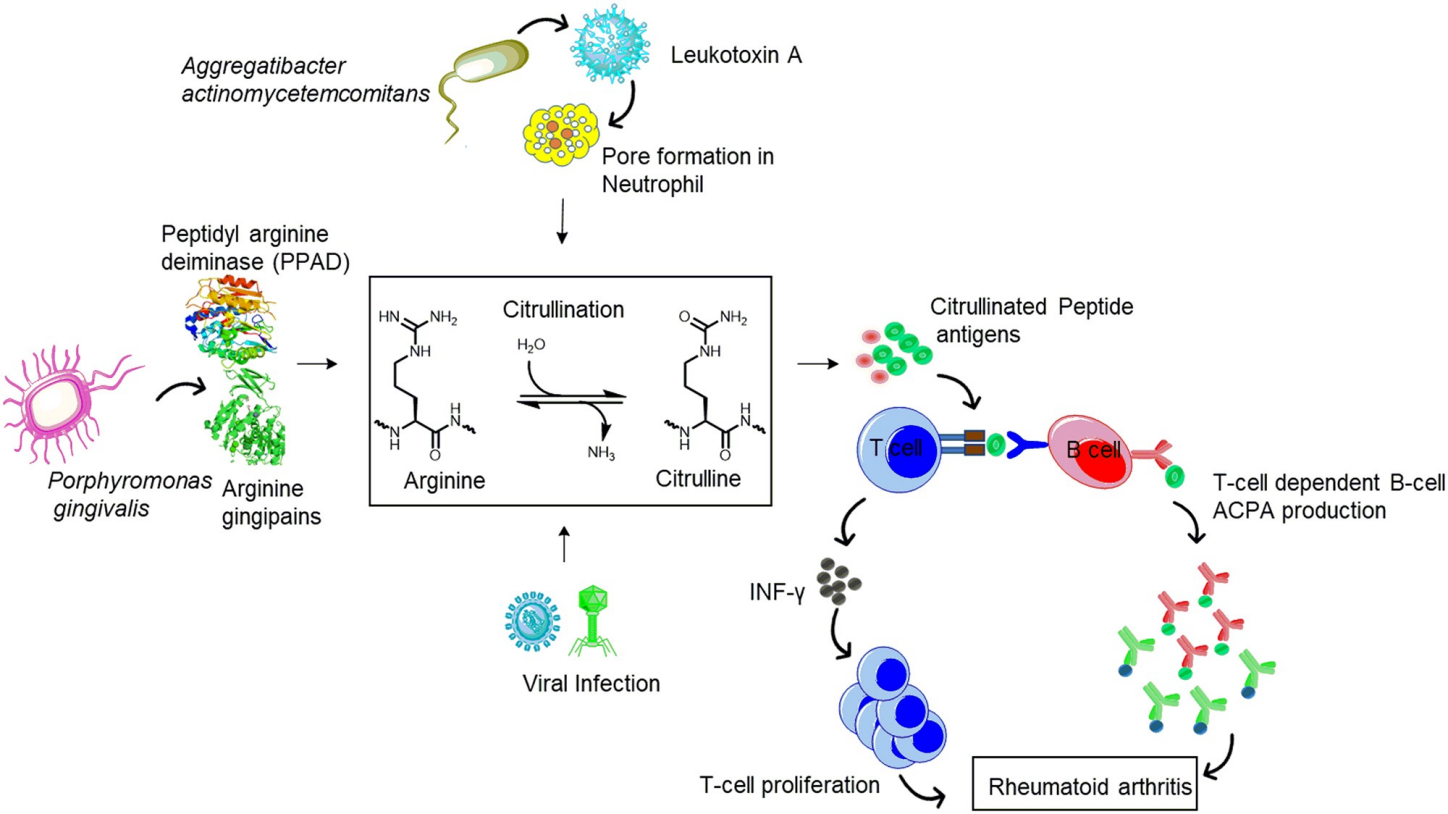
Schematic representation of citrullinated peptide in rheumatoid arthritis development.

Citrullination is a post-translational modification of arginine to citrulline catalyzed by peptidylarginine deiminase (PAD) during inflammation, apoptosis, and keratinization (Fig 1). Among the five PAD isotypes, PAD2 and PAD4 are thought to be associated with RA [3]. Three infectious agents, namely *Porphyromonas gingivalis*, *Aggregatibacter actinomycetemcomitans*, and Epstein-Barr virus (EBV), are believed to trigger citrullination in RA and thus render citrullinated peptide as potential arthritogenic neoantigens that lead to ACPA production (Fig 1) [8–12]. Indeed, citrullination also elevates the binding affinity of peptide autoantigens to *HLA-DRB1SE*, which is one of the risk factors for ACPA production [13]. The mechanism of pathogenesis is not fully understood. To date, it is confirmed that citrullinated peptide autoantigens induce T-cell-mediated B cell activation [14,15]. This action leads to ACPA production by hyper-reactive B cells and activates pro-inflammatory mediators, which subsequently cause joint inflammation and erosion. Different citrullinated epitopes, including fibrinogen and vimentin, have been reported in the synovium as a target of RA-specific autoantibodies [3,16,17]. Notably, blood levels of ACPA are reported to be lower than in synovial fluid [17].

Modifications in autoantigens represent a trigger for the generation of autoantibodies [18]. In the pathogenesis of RA in particular, citrullination of proteins has been shown to be a critical process, and there are a few reports underlying the role of protein mutations in citrullination and a consequent ACPA activation. Bang et al. studied the role of mutated and citrullinated vimentin (MCV) in RA and proposed it as a diagnostic and prognostic marker for RA. According to this study, antigenic properties of vimentin were determined by both mutation and citrullination [18].

Next, Takizawa et al. confirmed the role of fibrinogen as a soluble ACPA epitope [3]. They also reported on the effect of mutations on ACPA-fibrinogen interactions. Indeed, dysfibrinogenaemia is often associated with the mutation of position 16 of fibrinogen leading to impaired release of fibrinopeptide A (FPA) [3]. The replacement of a basic arginine with neutral citrulline at this position may hamper the binding of thrombin. Thus protected from polymerisation and made stable as a soluble antigen, citrullinated fibrinogen may become antigenic as a modified form of the self-antigen, fibrinogen [3].

Here, we hypothesized that rationally designed citrullinated peptide epitopes can become valuable tools for diagnosing RA and monitoring disease progression. We tested our hypothesis by developing a series of 20 citrullinated peptides and screening these in a cohort of RA-positive individuals and controls.

## Methods

The study has been approved by Danish Information Security Council, in 2011; by Pernille Winther Christensen; permit no. 11/32421, granted to Statens Serum Institute as a sample collector. Consent for minors (age < 18) has been obtained from parents. Number of patients for the study has been stratified by power calculation with 90% power estimated for RA/control cohorts with over 28 patients each [19]. For disease scoring and sampling included in longitudinal study, the visit dates were within 10-day range from given time point.

BLAST was performed for identifying all mutated peptides as input files, using all non-redundant GenBank CDS translations+PDB+SwissProt+PIR+PRF excluding environmental samples from WGS projects, Program ID BLASTP 2.8 [20]. Web Link for accessing BLAST: https://blast.ncbi.nlm.nih.gov/Blast.cgi. Since synthetic peptides have been used as an input, no particular accession number has been used. BLAST has been conducted over entire sequence space of human proteins.

Peptide antigens were synthesized and characterized as described in our recent papers [21–23]. In house ELISA has been carried out as described, using sera samples from patient groups and controls [21]. Commercial ELISA kits have been obtained from Abnova, and used following the corresponding manufacturer’s protocols (CCP2, Abnova KA4877; RF, Abnova KA2913; ANA, Abnova KA0939). Optical read-out (A450) has been used to monitor binding of autoantibodies to antigens [24–26].

Repeated measurements have been applied to calculate intra- and inter-assay coefficients of variance (CV; S1 Table in S1 Appendix). For this study, 10 RA and 10 healthy control samples have been used. Thus, triplicate measurements (ELISA) for E1, E2 and E3 resulted in the intra-assay CV values of 6.7%, 6.4% and 6.8%, respectively. Intra-assay CV for CCP2, RF and ANA ELISA assay were as follows: 10.6%, 9.2% and 10.9%, respectively.

Triple independent assays have been carried out for calculating inter-assay CV values. This has been done using same samples as for the intra-assay CV calculation, see above. For E1, E2 and E3, inter-assay CV values were 2.2%, 3.1% and 2.2%, respectively. Intra-assay CV for CCP2, RF and ANA ELISA assay were as follows: 5.3%, 8.3% and 6.5%, respectively.

### Statistical analyses

Data distribution normality has been confirmed by Shapiro-Wilk normality test in R [19]. Differences were analyzed for statistical significance with OLS and ANOVA in R. A P value of less than 0.05 was considered statistically significant.

## Results

To determine the novel reactivities of autoantibodies in RA patients, we developed 20 citrullinated peptide epitopes and applied them as antigens in ELISA. Sequences of the designed antigens, following our recently reported strategy [21], are shown in Table 1. The epitopes originates from five previously described proteins with relevance to RA pathogenesis: fibrinogen (E1, E4, and E6), vimentin (E9 and E10), histone 3 (E11-E15), collagen (E16), and filaggrin (E17-E20; Table 1). These proteins are involved in connective tissue onto- and neogeneses, and are therefore often affected in RA.

**Table 1:** Citrullinated peptide epitopes used in this study [2,1,27–3,1].

Epitope#	Sequence	Protein origin	Comments
E1	HHP GIA EFP S(Cit)G KSS SYS KQF	fib	Sequence reported in [27]
E2	HHP GIA EFP S(Cit)G KSY SYS KQF		Mutated epitope E1
E3	HGP GIA EFP S(Cit)G PSY SYS KQF		Mutated epitope E1
E4	AEGGGV(Cit)GPRVVE	fib	Sequence reported in [27]
E5	ASSGGV(Cit)GPRIVE		Mutated epitope E4
E6	KDLLPS(Cit)D(Cit)QHLPLIK	fib	Sequence reported in [27]
E7	QMRMELE(Cit)PGGNEIT(Cit)GGSTSYG	fib	Sequence reported in [27]
E8	NVSPGT(Cit)(Cit)EYHTEK	fib	Sequence reported in [27]
E9	ST(Cit)SVSSSSY(Cit)(Cit)MFGG	vim	Sequence reported in [28]
E10	VYAT(Cit)SSAV(Cit)L(Cit)SSVP	vim	Sequence reported in [28]
E11	A(Cit)TKQTA(Cit)KSTGGKAP	His	Citrullinated fragment of human histone 3 [29]
E12	AA(Cit)KSAPSTGGVKKPH	His	Citrullinated fragment of human histone 3 [29]
E13	Y(Cit)PGTVAL(Cit)EIKKYQKS	His	Citrullinated fragment of human histone 3 [29]
E14	LI(Cit)KLPFQ(Cit)LV(Cit)EIAQDFK	His	Citrullinated fragment of human histone 3 [29]
E15	LCAIHAK(Cit)VTIMPKDI	His	Citrullinated fragment of human histone 3 [29]
E16	A(Cit)GLTG(Cit)PGDA	col	Citrullinated fragment of collagen [30]
E17	SHQEST(Cit)G(Cit)S(Cit)GRSGRSGS	fil	Citrullinated fragment of filaggrin [31]
E18	T(Cit)GRS	fil	Citrullinated fragment of filaggrin [31]
E19	T(Cit)G(Cit)S	fil	Citrullinated fragment of filaggrin [31]
E20	TRG(Cit)S	fil	Citrullinated fragment of filaggrin [31]

^a^ fib = fibrinogen; vim = vimentin; His = histone; col = collagen; fil = filaggrin.

Importantly, fibrinogen can be mutated in RA patients [32]. This fact gives rise to the hypothesis that an amino acid mutation and a citrulline modification of arginine can together act as an epitope for the RA-associated autoantibody. Using the Protein databank, we identified common fibrinogen epitopes that are mutated in RA; this effort led to the E2, E3, and E5 sequences. Notably, the potential citrullinated and/or mutated epitopes are not limited to those given in Table 1. Other relevant sequences might be discovered in the future, as more sequences for proteins in RA subjects are collected.

**Fig 2:**
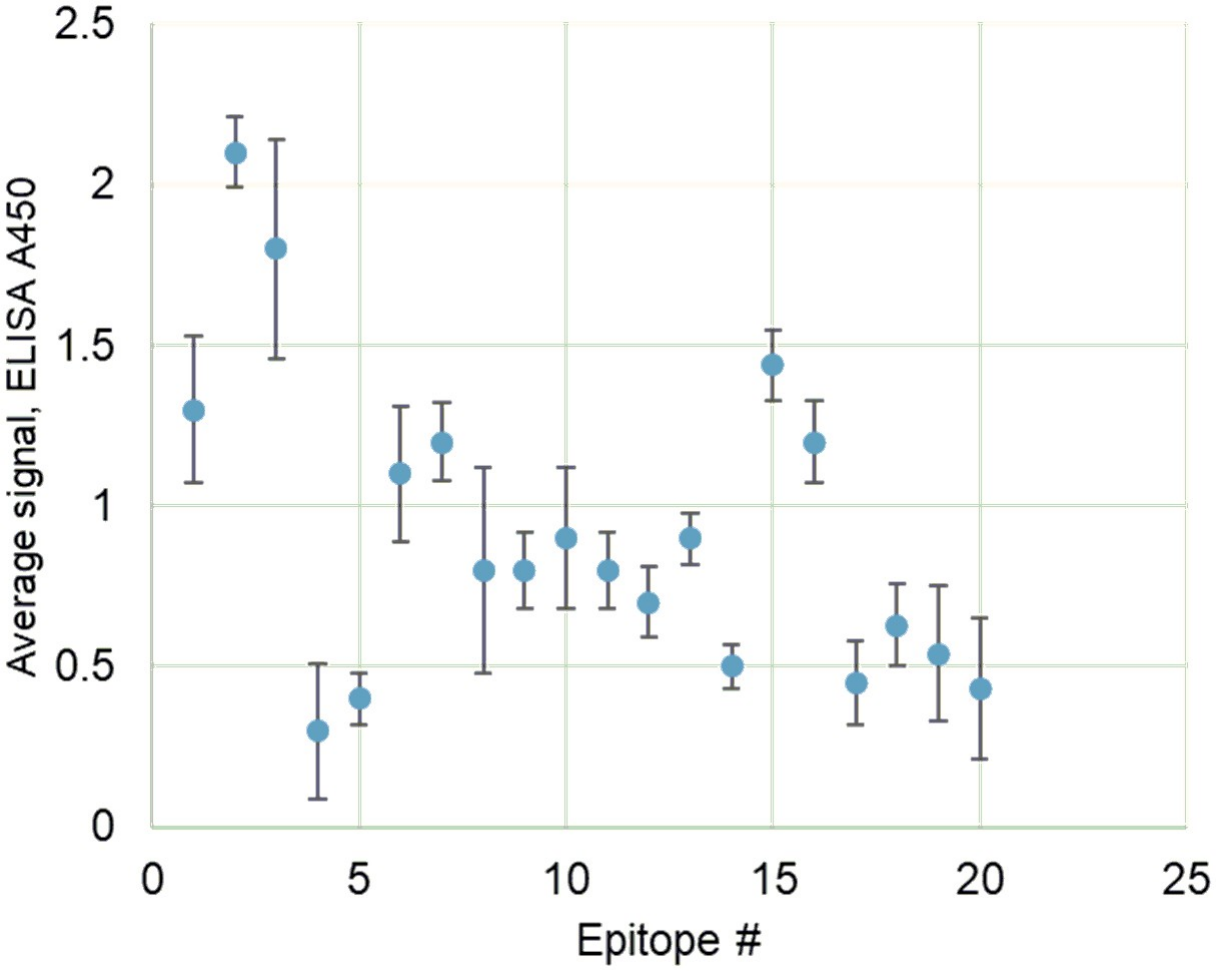
Mean response to epitopes E1-E20 in RA samples, n = 70. Each sample has been analyzed in duplicate, and in two independent ELISA experiments, with CV below 5% being considered as a reliable result.

Peptide epitopes were purchased from a commercial supplier (synthesized by solid-phase peptide synthesis [22,23]; HPLC purified fractions with purity > 90%). The initial screening was performed using 70 RA samples (RA) at disease onset; 87% of RA patients were female, 100% were Caucasian, and the mean age at sampling was 41 years (range: 28–56). The mean disease activity score (DAS28) for these patients was four (range: 2–8). The results for ELISA using epitopes E1-E20 are shown in Fig 2; individual data points are given in S2 Appendix. Our initial screening allowed us to identify the three most potent epitopes (E1-E3), all derived from fibrinogen. These epitopes showed elevated levels of antibodies that correlated with a higher ACR score (p < 0.00001 at 95% confidence interval; determined by one-way analysis of variance [ANOVA] for normally distributed data; all tests conducted in R).

To further study the reactivity to citrullinated fibrinogen epitopes E1, E2, and E3 in RA, we collected a longitudinal cohort of RA samples; the cohort’s features are provided in Table 2 and in S3 Appendix. In brief, we included 30 RA subjects in the study. They were monitored over 5 years with intervals of 3–7 month between visits (samples provided by SSI, Denmark). The majority were female, and the mean age at diagnosis was 29 years (range: 22–44); 90% were Caucasian and 10% were Asian. Written consent was obtained from all patients; all their data for this study were depersonalized.

**Table 2:** Demographical and clinical features of RA patients and controls used in this study.

Feature/Patient group	RA (n = 30)	SLE (n = 30)	HC (n = 30)
% females	83	78	83
Age at diagnosis, mean/range	29 (22–44)	26 (16–38)	30 (21–44)
Disease activity score DAS28 mean/range**	5 (2–8)	6 (0–11)	n/a
CDAI	18 (1.3–53)	n/a	n/a
SDAI	23 (2.4–59)	n/a	n/a
Caucasian, %	90	90	90
Afro-American, %	0	0	0
Pacific Islander, %	0	0	0
Asian, %	10	10	10
**Serology**:			
RF positive, %	17	0	9
ACPA positive, CCP2, %	30	0	0
ANA positive, %	21	60	3
MMP3 positive, %	16	2	0
ESR mean/range	6.3 (2.1–11.3)	6.5 (1.4–9.3)	4.4 (3.2–5.7)
C4 mean/range	17 (10–29)	21 (11–32)	30 (24–42)
Treatment NSAIDS, %	54	77	0
Treatment, steroids, %	77	45	0
Treatment, other %***	33	33	11
**Longitudinal samples, 5Y change (Δ)**			
ΔDAS28/ΔSLEDAI	-1	-2	-
ΔCDAI (RA samples)	-7	n/a	n/a
ΔSDAI (RA samples)	-9	n/a	n/a
ΔRF	-1	0	-
ΔCCP2	0	0	-
ΔANA	0	-1	-
ΔMMP3	-2	0	-
ΔESR	+4.0	-4.1	-
ΔC4	-4.5	-5	-
ΔTreatment NSAIDS, %	-12	-21	-
ΔTreatment, steroids, %	-23	-31	-
ΔTreatment, other %	-34	-21	-

** Disease activity (DAS28 and SLEDAI for RA and SLE, respectively) scores have been applied. Besides, CDAI (clinical disease activity index), and SDAI (simplified disease activity index) were applied to RA cohort. MMP3 = matrix metalloproteinase-3; ESR = erythrocyte sedimentation rate; NSAIDS = non-steroidal anti-inflammatory drug. ELISA was conducted following our published procedure [21]. RF positivity is a hallmark of over 50% of RA cases [5]. Besides, the commercial ACPA test (CCP2) was applied. We also included antinuclear antibodies (ANA), determined by Hep2 cellular assay, as a non-specific biomarker for autoimmune diseases in this control study. MMP3, ESR, and C4 were other common serological markers that we conducted (Table 1) [2–5].

*** Other treatment included biological drugs adalimumab and infliximab for RA, and belimumab for SLE.

At each visit, a patient’s disease activity was scored following the disease activity (DAS28) score, with 0 indicating no symptoms and 10 representing the greatest disease burden [7]. Besides, CDAI (clinical disease activity index), and SDAI (simplified disease activity index) were applied to RA cohort. Our serological testing included ACR/EULAR recommended tests: ACPA, RF, ANA, and ELISA using our new epitopes. Our RA cohort included “poor responders” to the initial treatment with MTX [6,7,33]. In this cohort, 7% patients experienced flare after 12-month treatment, and 67% responded poorly to treatment at 60-month time point (see S3 Appendix).

Control samples were matched to the RA cohort, with 30 systemic lupus erythematosus (SLE) patients—83% female and mean age at diagnosis of 26.0 years (range: 16–38)—monitored over 5 years, with intervals of 2–7 months (S4 Appendix). 90% of SLE patients were Caucasian, and 10% were Asian. Besides, we included 30 matched healthy controls, 83% female with a mean age of 30 years (range: 21–44; S5 Appendix). SLE subjects were scored at each sampling with SLEDAI score, where 0 represents the lowest disease activity and 30 indicates maximal disease burden. The serology for SLE and healthy controls included ACPA, RF, ANA, MMP3, and ELISA using our epitopes. Data analysis was performed using descriptive statistics (R), ANOVA, and ordinary least squares (OLS).

**Fig 3:**
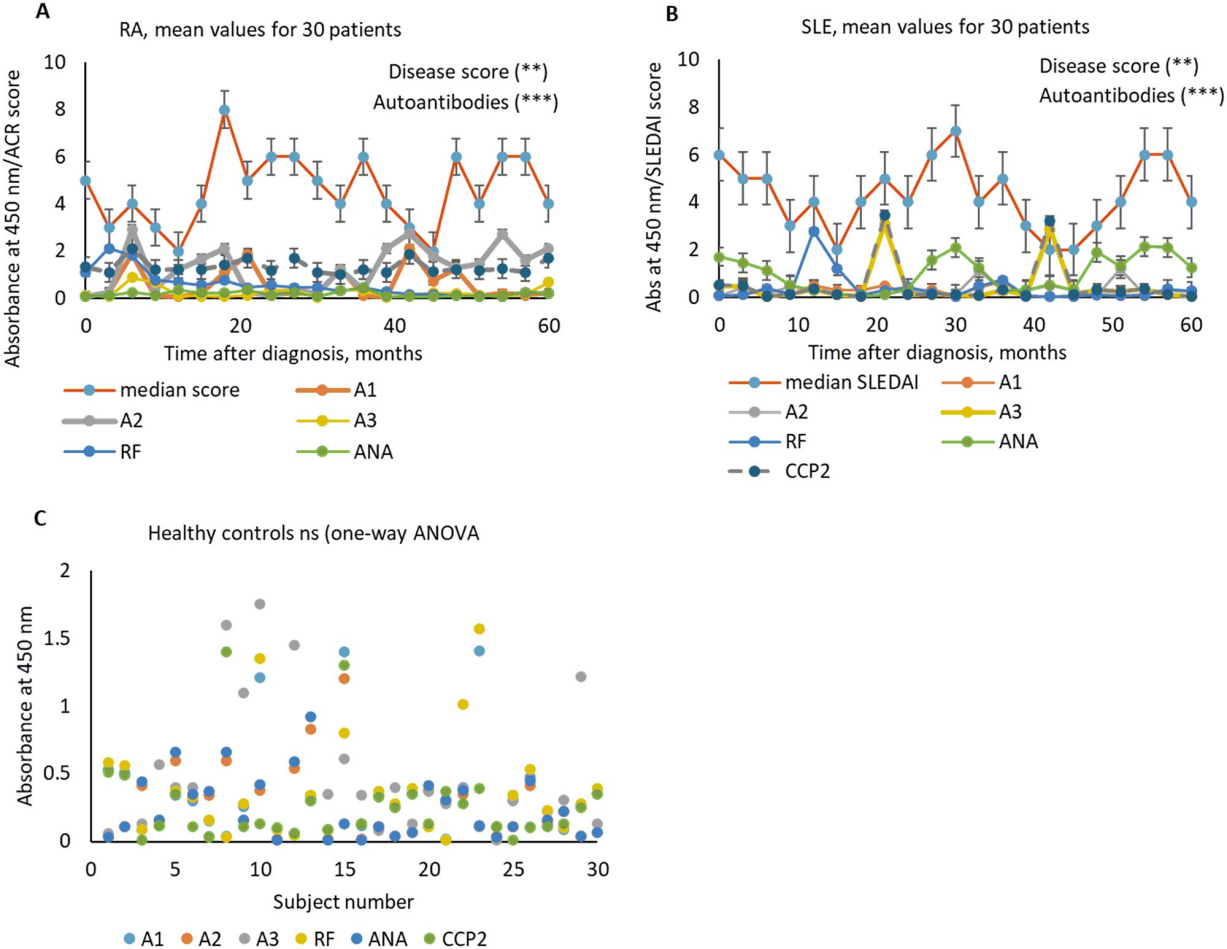
Longitudinal study of patients across a set of antigens: RA (A), SLE (B) and healthy controls (C).

The results are shown in Table 2 and Fig 3. Individual data points are given in S6 Appendix. Median disease score for RA increased after 12-month treatment, data that indicated a flare in over 7% of patients. Two subsequent flares were observed between 34 and 45 months after initiating treatment. Overall, 67% RA subjects failed to respond to applied treatment. Control SLE patients flared between 30 and 55 months after initiating treatment.

In RA, a-E3 levels remained rather low during the entire treatment course. A-CCP2 remained elevated without a statistically significant sensitivity to flares. ANA was slightly elevated during flares. Sensitivity to flares was observed for levels of antibodies against E1 and E2 epitopes. Notably, antibody levels to the original E1 and its mutated version differentially correlated with the disease activity score. Anti-E1 was elevated during flare periods, whereas a-E2 increased approximately 2 months prior to a flare and decreased with reduced disease activity. ACR/EULAR scores correlated linearly with a-E2 (p < 0.001 with OLS). For CCP2 and ACPA, the correlation was not statistically significant, with p = 0.56 with OLS for ACPA-ACR/EULAR scores.

In SLE (S7 Appendix), ANA was elevated during flares, data that are consistent with previous reports [34]. RF was elevated approximately 12 months post-treatment, most likely as a response to therapy [35]. ACPA and anti-E3 were elevated prior to flares. Anti-E1 and anti-E2 were not sensitive to flares in SLE. Remarkably, the fibrinogen peptide epitope also recognizes autoantibodies in SLE, and this recognition occurs in a different manner compared to RA. This phenomenon unifies RA and SLE as rheumatic autoimmune diseases with a certain overlap in pathology [36], although there is a difference in antibody-epitope recognition at the molecular level [37]. It is also remarkable that CCP2 and ACPA are elevated in some SLE samples. However, there is no correlation of their levels with SLEDAI (CCP2-SLEDAI OLS p = 0.23).

In healthy controls (S5 Appendix), there was no statistically significant difference in responses across antigens; the responses were low in more than 86% of samples (p = 0.21 by one-way ANOVA, all groups). There were few outlier samples (less than 10%) that had elevated anti-E3, RF, and ACPA (Fig 3C).

We correlated RA to SLE and RA to healthy controls for anti-E2 levels using ANOVA; in both cases, p < 0.00001. This finding confirms the relevance of anti-E2 and the epitope to RA. The RA-SLE correlation for ACPA was significant (p = 0.03); however, the RA to healthy control correlation was not statistically significant (p = 0.56). These data crucially demonstrate that the E2 assay has a superior specificity.

**Fig 4:**
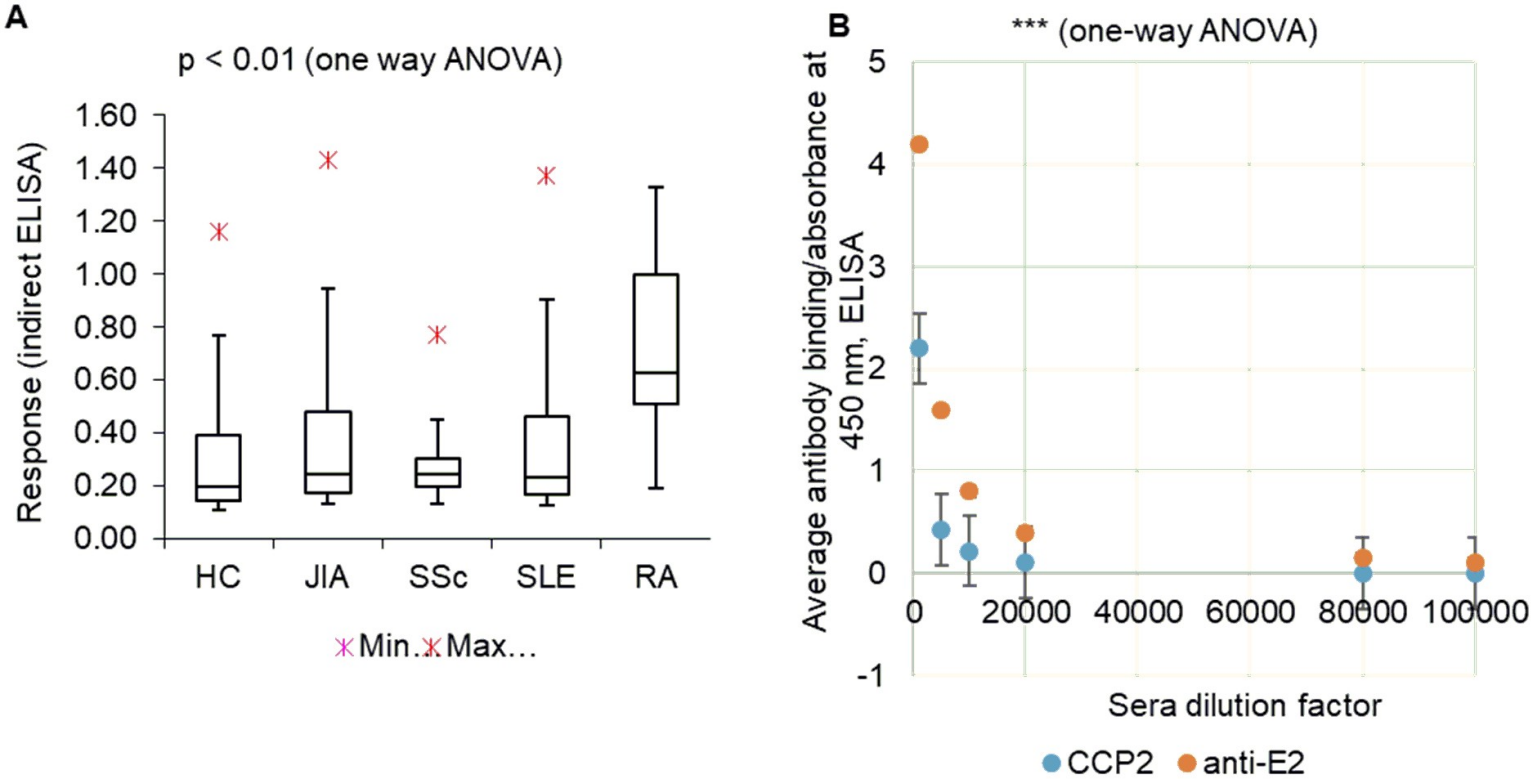
Clinical (A) and analytical (B) sensitivity study of anti-E2 test vs controls. HC = healthy control, JIA = juvenile idiopathic arthritis, SSc = systemic sclerosis, SLE = systemic lupus erythematosus, RA = rheumatoid arthritis. (B) Mean reactivity for 20 samples at onset is given.

Next, we focused on the disease specificity of the tests. We extended the control samples to the following populations: juvenile idiopathic arthritis (JIA; n = 54), systemic sclerosis (SSc; n = 80), SLE (n = 400), and healthy controls (n = 60). Anti-E2 was statistically significantly elevated in RA samples (p < 0.01 by one-way ANOVA for normally distributed data; Fig 4 and S8 Appendix). Among controls, JIA had the highest number of ani-E2-elevated samples. Analytical sensitivity determined by sera titration experiment was 1:100,000 sera dilution (determined as an average signal for 20 RA samples), which was 5-fold superior compared to the existing ACPA test (Fig 4B) [38, 39]. We performed receiver operating characteristic (ROC) analysis for E2 and CCP2 tests. This analysis provided true positive rate (TPR), false positive rate (FPR), and area under the curve (AUC) values for E2 of 0.86, 0.08, and 0.92, respectively. For CCP2, the values were 0.68 (TPR), 0.24 (FPR), and 0.78 (AUC) (S8 Appendix).

**Fig 5:**
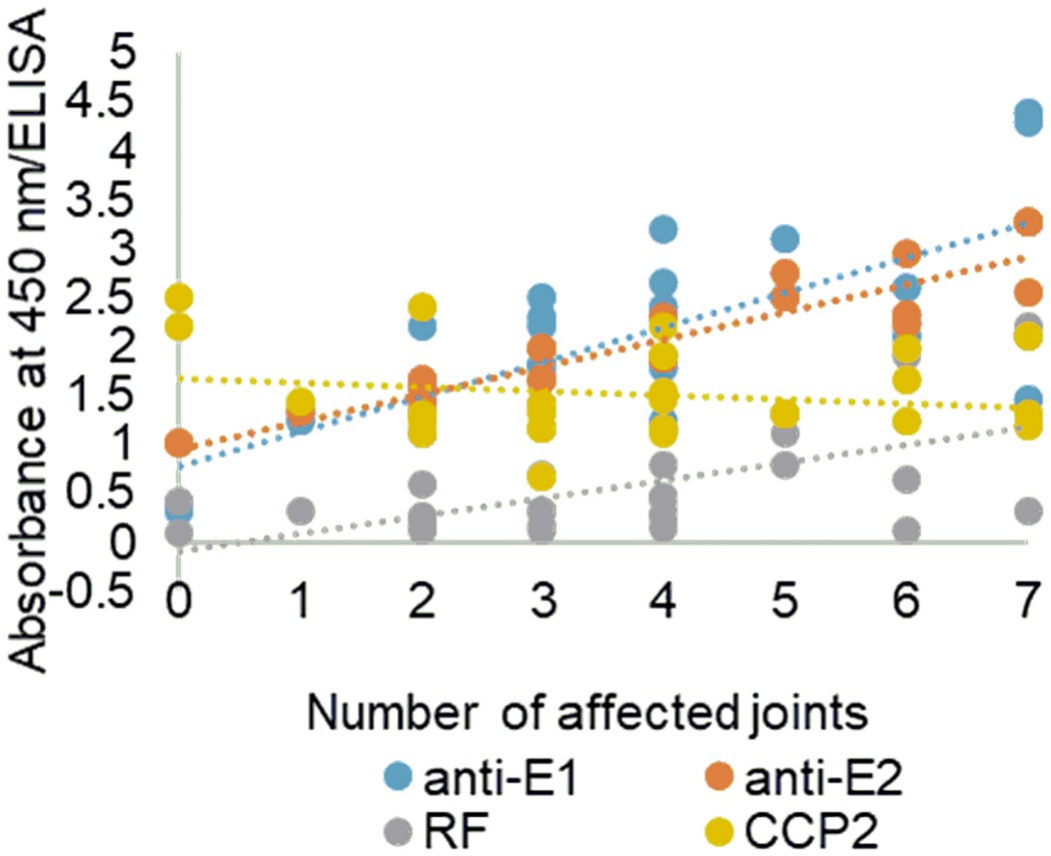
Correlation of anti-E1, anti-E2, ACPA and RF reactivity with number of inflamed joints in RA patients (n = 30; cohort data given in Table 2).

Damage to multiple joints is an important feature of RA progression. Therefore, we performed a statistical analysis by linear regression of the autoantibody reactivities (E2, CCP2 ACPA, and RF) versus the number of joints affected in RA patients. First, we confirmed that the data was normally distributed. Then, using OLS in R, we examined correlations of affected joint number with anti-E1, anti-E2, CCP2 and RF levels (Fig 5; S9 Appendix). Anti-E2 had the strongest linear correlation with the number of affected joints, followed by anti-E1 and commercial CCP2 ACPA. For RF and CCP2, the correlation was not statistically significant.

## Discussion

Recent investigations into the molecular nature of ACPA are elucidating how these antibodies are optimized to recognize citrullinated proteins and peptides. Once such study found ACPA-IgA in RA patients to be 15–20 kDa heavier than non-autoreactive IgG, with the added mass being predominantly from N-glycans [40]. As more structural information is revealed, the task of rationally designing peptide epitopes for ACPA might become clearer. In parallel, efforts to identify new endogenous protein and peptide epitopes from RA patients are ongoing. A recent study applied matrix-assisted laser desorption/ionization-time of flight (MALDI-TOF) to examine synovial fluid from RA patients. It revealed 182 citrullinated peptides, most of which were not previously reported [41].

Rationally designed peptides have been used by others in order to investigate the recognition specificity of the original epitopes or develop novel epitopes with favorable characteristics for diagnostics. One group tested four citrullinated peptides based on fibrinogen for RA diagnosis and found the best candidate, Cit74, has 64–65% sensitivity in CCP2-positive RA sera [42]. Another study designed 14 cyclic peptides by truncation to a known filaggrin epitope and compared reactivity with the original sequence [43]. The study showed that cyclization is crucial for retaining activity with shorter peptides. Additionally, the presence of a glycine c-terminal next to citrulline is important, which is consistent with our most active peptide epitope.

In the RA cohort that we have studied herein, patients experienced flares after 12-month treatment. This differentiates them from a typical RA disease course [6,7,33]. One particular issue leading to the flare is a poor response to MTX in our RA cohort. Moreover, 67% of the patients failed on repeated treatments with MTX, DMARD and biological drugs as well. Rationally selected epitope E2 showed advantageous properties in detecting flares in these patients compared to commercial tests. This feature of E2 makes it potent in improved management of “poor responder” RA patients [6,7,33].

In conclusion, we propose a new citrullinated peptide epitope for diagnosis of RA-related autoantibodies and to predict flares. In our retrospective study, we confirmed the high reactivity of antibodies to a fibrinogen-derived citrullinated epitope that correlated with both the disease activity and the number of affected joints. Compared to existing tests, our developed anti-E2 has an advantage of robustness and relevance in a longitudinal study. The complexity of RA pathogenesis implies a potentially high number of relevant auto-antigens, with citrullinated peptides being just a portion of the potential variety. We believe that our results will inspire a continued search for improved biomarkers to effectively diagnose and manage RA, while also providing new insights into the disease pathology at the molecular level.

## Supporting information

S1 Appendix Replicated measurements, RA and HC samples, and CV values. S1 Table. Triplicate results for ELISA: RA (samples 1–10) and HC (samples 11–20).
(PDF)

S2 Appendix ELISA results for screening of peptide epitopes. S2 Table. A450 data for screening of epitopes E1-E20, using RA cohort, N = 70.
(PDF)

S3 Appendix RA cohort information. S3 Table. Onset data for RA subjects, longitudinal cohort (N = 30). S4 Table. Data for RA subjects 60 months after treatment.
(PDF)

S4 Appendix SLE cohort information. S5 Table. Data for longitudinal SLE subjects at onset, N = 30. S6 Table. Data for SLE subjects 60 months after treatment.
(PDF)

S5 Appendix Healthy controls—Information. (PDF)

S6 Appendix Longitudinal study, RA cohort, individual data points. S8 Table. Mean A450 values, RA cohort (N = 30). S9 Table. Data for longitudinal study of RA cohort (N = 30).
(PDF)

S7 Appendix Mean A450 and individual A450 values, SLE cohort. S10 Table. Mean A450 values, SLE cohort (N = 30). S11. Individual data points for longitudinal study, SLE cohort.
(PDF)

S8 Appendix Individual values for ELISA of healthy and diseased controls; ROC analysis. S12 Table. E2 ELISA results (A450 values), for individual samples: healthy controls (HC), JIA, SSc, SLE and RA. S14 Table. Individual data points for serum dilution study, 20 RA patients. S1 Fig. Results of ROC for E2 (A) and CCP2 (B).
(PDF)

S9 Appendix Joint count correlation study; data for individual patients. S13. Table. Data for RA individuals applied in joint count correlation study.
(PDF)
