# Supplementary material for: Antibodies to synthetic citrullinated peptide epitope correlate with disease activity and flares in rheumatoid arthritis
Source: PLoS One. 2020 Apr 23;15(4):e0232010. doi: 10.1371/journal.pone.0232010 (PMC7179858; doi:10.1371/journal.pone.0232010)
Supplement: S1 Appendix — S1 Table. Triplicate results for ELISA: RA (samples 1–10) and HC (samples 11–20). (PDF) [file pone.0232010.s001.pdf]

S1 Appendix. Replicated measurements, RA and HC samples, and CV values

S1 Table. Triplicate results for ELISA: RA (samples 1-10) and HC (samples 11-20)

Different plates<br>Sample no	E1			CV, %	E2			CV, %	E3			CV, %
1	0,09	0,10	0,09	*2,61*	0,14	0,14	0,13	*3,45*	0,20	0,21	0,20	*2,32*
2	0,11	0,08	0,10	*11,41*	0,07	0,10	0,08	*14,99*	0,06	0,08	0,06	*15,83*
3	0,13	0,10	0,11	*11,36*	0,05	0,05	0,05	*1,91*	0,18	0,17	0,17	*2,72*
4	0,13	0,10	0,12	*10,69*	0,09	0,06	0,07	*13,90*	0,08	0,07	0,07	*7,64*
5	0,12	0,10	0,14	*13,61*	0,09	0,08	0,09	*7,23*	0,21	0,20	0,20	*2,32*
6	0,08	0,08	0,07	*4,99*	0,14	0,12	0,13	*6,28*	0,06	0,07	0,06	*6,75*
7	0,06	0,07	0,06	*6,36*	0,10	0,10	0,09	*4,37*	0,14	0,13	0,15	*5,83*
8	0,13	0,12	0,12	*3,82*	0,10	0,10	0,11	*5,77*	0,07	0,07	0,08	*6,10*
9	0,10	0,10	0,11	*4,82*	0,08	0,08	0,09	*4,45*	0,11	0,10	0,14	*14,57*
10	0,13	0,12	0,13	*3,72*	0,12	0,10	0,12	*8,32*	0,11	0,11	0,10	*4,42*
11	0,11	0,13	0,11	*8,08*	0,10	0,10	0,11	*5,43*	0,17	0,16	0,18	*4,80*
12	0,11	0,11	0,11	*0,00*	0,10	0,08	0,10	*7,80*	0,13	0,10	0,13	*11,79*
13	0,12	0,10	0,11	*7,82*	0,09	0,09	0,09	*1,37*	0,23	0,20	0,23	*6,43*
14	0,07	0,06	0,07	*6,38*	0,05	0,05	0,05	*2,77*	0,15	0,15	0,14	*3,21*
15	0,08	0,08	0,10	*9,05*	0,08	0,10	0,08	*9,04*	0,13	0,13	0,12	*3,72*
16	0,13	0,12	0,11	*6,80*	0,11	0,10	0,11	*4,42*	0,08	0,08	0,08	*1,17*
17	0,08	0,08	0,09	*5,42*	0,08	0,07	0,08	*5,46*	0,14	0,11	0,13	*9,85*
18	0,09	0,10	0,09	*5,34*	0,10	0,10	0,08	*10,64*	0,03	0,02	0,03	*20,09*
19	0,10	0,09	0,09	*6,63*	0,09	0,09	0,10	*5,05*	0,10	0,10	0,10	*0,00*
20	0,11	0,12	0,11	*4,16*	0,11	0,11	0,10	*5,34*	0,07	0,07	0,08	*6,10*
**CV, mean, %**				**6,7**				**6,4**				**6,8**

Different plates<br>Sample no	RF			CV, %	ANA			CV, %	ACPA			CV, %
1	1,50	1,20	1,43	*9,3*	0,07	0,05	0,08	*19,6*	1,40	1,00	1,30	*13,8*
2	0,75	0,80	0,72	*4,4*	0,07	0,08	0,08	*3,2*	1,30	1,20	1,30	*3,7*
3	0,91	0,90	0,82	*4,6*	0,08	0,08	0,07	*4,0*	1,40	1,30	1,20	*6,3*
4	1,40	1,30	0,79	*23,0*	0,09	0,10	0,07	*12,1*	1,20	1,20	0,98	*9,2*
5	1,50	1,55	1,51	*1,4*	0,08	0,09	0,06	*15,2*	1,10	1,20	1,10	*4,2*
6	0,16	0,26	0,15	*26,1*	0,07	0,08	0,08	*10,0*	1,20	1,30	1,10	*6,8*
7	0,49	0,44	0,45	*4,7*	0,09	0,09	0,09	*1,9*	0,95	0,98	1,40	*18,5*
8	1,40	1,43	1,44	*1,2*	0,08	0,07	0,07	*4,4*	1,30	1,20	1,10	*6,8*
9	2,10	2,10	1,90	*4,6*	0,07	0,08	0,09	*12,6*	0,97	1,40	1,60	*19,9*
10	0,69	0,66	0,62	*4,4*	0,07	0,07	0,06	*12,7*	1,40	1,00	0,97	*17,4*
11	0,06	0,05	0,05	*5,9*	0,07	0,06	0,06	*10,3*	0,07	0,06	0,05	*12,1*
12	0,07	0,08	0,06	*11,6*	0,06	0,07	0,07	*3,7*	0,08	0,07	0,06	*9,8*
13	0,06	0,05	0,07	*15,7*	0,06	0,06	0,06	*3,8*	0,06	0,05	0,06	*7,1*
14	0,05	0,05	0,07	*15,4*	0,06	0,08	0,06	*17,0*	0,06	0,04	0,06	*13,9*
15	0,04	0,06	0,08	*24,8*	0,05	0,08	0,06	*16,3*	0,08	0,06	0,06	*12,8*
16	0,07	0,07	0,04	*24,6*	0,06	0,09	0,08	*18,5*	0,06	0,07	0,07	*11,2*
17	0,06	0,05	0,04	*18,2*	0,07	0,07	0,08	*6,1*	0,06	0,05	0,05	*3,8*
18	0,05	0,05	0,05	*0,9*	0,08	0,07	0,07	*5,7*	0,07	0,05	0,05	*14,1*
19	0,07	0,07	0,07	*3,2*	0,07	0,07	0,07	*3,1*	0,06	0,08	0,06	*13,3*
20	0,06	0,06	0,05	*8,4*	0,08	0,08	0,08	*3,1*	0,95	1,30	1,30	*13,9*
**CV, mean, %**				**10,6**				**9,2**				**10,9**

Same plate<br>Sample no	E1			CV, %	E2			CV, %	E3			CV, %
1	0,09	0,09	0,09	*1,85*	0,14	0,14	0,14	*0,00*	0,20	0,20	0,20	*0,00*
2	0,11	0,11	0,11	*0,00*	0,07	0,07	0,08	*6,80*	0,06	0,07	0,06	*6,75*
3	0,13	0,13	0,12	*3,72*	0,05	0,04	0,05	*9,39*	0,18	0,18	0,18	*0,00*
4	0,13	0,13	0,12	*3,72*	0,09	0,09	0,09	*0,53*	0,08	0,08	0,07	*6,79*

5	0,12	0,12	0,13	*3,82*	0,09	0,09	0,09	*2,06*	0,21	0,21	0,20	*2,28*
6	0,08	0,08	0,08	*2,17*	0,14	0,14	0,13	*3,45*	0,06	0,06	0,06	*1,55*
7	0,06	0,06	0,06	*3,07*	0,10	0,10	0,10	*0,00*	0,14	0,13	0,14	*3,45*
8	0,13	0,12	0,12	*3,82*	0,10	0,10	0,10	*1,91*	0,07	0,07	0,07	*0,67*
9	0,10	0,10	0,10	*0,47*	0,08	0,08	0,09	*5,14*	0,11	0,11	0,11	*0,00*
10	0,13	0,13	0,13	*0,00*	0,12	0,11	0,11	*4,16*	0,11	0,11	0,11	*0,00*
11	0,11	0,12	0,12	*4,04*	0,10	0,10	0,10	*1,43*	0,17	0,17	0,18	*2,72*
12	0,11	0,11	0,11	*0,00*	0,10	0,10	0,10	*1,91*	0,13	0,13	0,13	*0,00*
13	0,12	0,12	0,11	*4,04*	0,09	0,09	0,09	*1,37*	0,23	0,22	0,22	*2,11*
14	0,07	0,07	0,07	*2,05*	0,05	0,04	0,05	*11,66*	0,15	0,15	0,16	*3,07*
15	0,08	0,08	0,09	*4,97*	0,08	0,09	0,08	*5,99*	0,13	0,13	0,13	*0,00*
16	0,13	0,13	0,13	*0,00*	0,11	0,10	0,10	*4,56*	0,08	0,09	0,08	*5,14*
17	0,08	0,08	0,09	*5,42*	0,08	0,08	0,08	*2,40*	0,14	0,14	0,13	*3,45*
18	0,09	0,09	0,09	*0,53*	0,10	0,10	0,10	*0,00*	0,03	0,03	0,03	*4,56*
19	0,10	0,10	0,10	*0,00*	0,09	0,09	0,09	*0,00*	0,10	0,10	0,10	*0,00*
20	0,11	0,11	0,11	*0,00*	0,11	0,11	0,11	*0,00*	0,07	0,07	0,07	*0,67*
**CV, mean, %**				**2,2**				**3,1**				**2,2**

Sampe plate<br>Sample no	RF			CV, %	ANA			CV, %	ACPA			CV, %
1	1,50	1,35	1,43	*4,3*	0,07	0,06	0,07	*7,1*	1,40	1,20	1,23	*6,9*
2	0,75	0,75	0,72	*1,9*	0,07	0,07	0,07	*2,0*	1,30	1,33	1,31	*0,9*
3	0,91	0,90	0,91	*0,5*	0,08	0,09	0,08	*6,8*	1,40	1,22	1,20	*7,1*
4	1,40	1,33	1,39	*2,3*	0,09	0,09	0,09	*2,7*	1,20	1,20	1,20	*0,0*
5	1,50	1,43	1,44	*2,1*	0,08	0,08	0,07	*5,7*	1,10	1,10	1,10	*0,0*
6	0,16	0,17	0,16	*2,9*	0,07	0,07	0,07	*3,4*	1,20	1,20	1,11	*3,6*
7	0,49	0,44	0,45	*4,7*	0,09	0,09	0,09	*1,9*	0,95	0,94	0,87	*3,9*
8	1,40	1,40	1,44	*1,3*	0,08	0,09	0,08	*6,0*	1,30	1,30	1,22	*3,0*
9	2,10	2,10	2,00	*2,3*	0,07	0,07	0,12	*28,8*	0,97	0,96	0,89	*3,8*
10	0,69	0,71	0,77	*4,7*	0,07	0,07	0,06	*11,6*	1,40	1,44	0,52	*37,9*
11	0,06	0,06	0,05	*4,7*	0,07	0,07	0,06	*8,0*	0,07	0,07	0,05	*12,3*
12	0,07	0,08	0,06	*11,6*	0,06	0,06	0,07	*5,1*	0,08	0,07	0,06	*9,8*
13	0,06	0,06	0,07	*6,5*	0,06	0,07	0,06	*7,0*	0,06	0,06	0,06	*4,1*
14	0,05	0,05	0,05	*3,3*	0,06	0,08	0,06	*17,0*	0,06	0,06	0,06	*4,2*
15	0,04	0,04	0,05	*9,5*	0,05	0,08	0,06	*16,3*	0,08	0,07	0,06	*11,0*
16	0,07	0,07	0,07	*2,3*	0,06	0,09	0,08	*18,5*	0,06	0,07	0,07	*11,2*
17	0,06	0,05	0,05	*11,6*	0,07	0,09	0,09	*10,7*	0,06	0,05	0,05	*3,8*
18	0,05	0,04	0,05	*9,7*	0,08	0,08	0,08	*2,4*	0,07	0,07	0,07	*1,4*
19	0,07	0,06	0,05	*11,8*	0,07	0,07	0,07	*3,1*	0,06	0,06	0,06	*2,9*
20	0,06	0,06	0,05	*8,4*	0,08	0,08	0,08	*3,1*	0,95	0,99	0,92	*3,0*
**CV, mean, %**				**5,3**				**8,3**				**6,5**
