# Supplementary material for: Antibodies to synthetic citrullinated peptide epitope correlate with disease activity and flares in rheumatoid arthritis
Source: PLoS One. 2020 Apr 23;15(4):e0232010. doi: 10.1371/journal.pone.0232010 (PMC7179858; doi:10.1371/journal.pone.0232010)
Supplement: S2 Appendix — S2 Table. A450 data for screening of epitopes E1-E20, using RA cohort, N = 70. (PDF) [file pone.0232010.s002.pdf]

S2 Appendix. ELISA results for screening of peptide epitopes.

ELISA has been carried out as described in Methods. Randomly selected 5% of samples have been tested in triplicate using the same plate. According to chi2 statistical test, 5% have been a sufficient sampling to represent the entire cohort (54 out of 1400 total observations; chi-square statistic is 63.4188. The p-value is < 0.00001). CV values were within the range given in Methods section. Below, individual data points for all subjects are given.

S2 Table. A450 data for screening of epitopes E1-E20, using RA cohort, N = 70.

Pat.no/epitope#	E1	E2	E3	E4	E5	E6	E7	E8	E9	E10	E11	E12	E13	E14	E15	E16	E17	E18	E19	E20
**1**	1.52	1.93	2.03	0.32	0.17	1.06	0.86	0.75	1.10	0.89	1.10	0.16	1.10	1.10	0.95	1.59	0.50	0.68	0.60	0.46
**2**	1.44	0.93	1.59	0.24	0.30	1.06	0.90	0.86	0.71	0.41	0.70	0.57	0.72	0.72	0.72	1.14	0.46	1.18	0.49	0.27
**3**	1.40	2.22	1.95	0.38	0.28	1.10	1.36	0.68	0.71	0.80	1.10	0.79	0.99	0.99	2.20	1.33	0.60	0.70	0.65	0.42
**4**	1.49	2.01	1.82	0.36	0.17	0.89	1.08	0.83	0.68	0.94	0.74	1.00	0.96	0.96	1.58	0.81	0.49	0.90	0.56	0.44
**5**	1.24	2.91	2.41	0.33	0.48	0.74	1.02	0.57	1.31	0.74	0.50	0.28	0.74	0.74	2.08	1.22	0.36	0.53	0.42	0.22
**6**	1.25	2.26	2.17	0.45	0.10	0.81	0.99	0.49	0.72	1.56	1.31	0.84	1.09	1.09	1.70	1.11	0.29	0.49	0.34	0.49
**7**	1.33	2.02	1.80	0.14	0.41	1.10	1.26	0.77	1.08	0.52	0.10	0.63	0.89	0.89	2.42	1.04	0.40	0.67	0.33	0.55
**8**	1.32	1.91	1.76	0.60	0.69	1.31	0.73	0.64	0.70	0.78	0.98	0.30	0.93	0.93	0.68	0.82	0.64	0.45	0.59	0.56
**9**	1.52	1.88	1.75	0.45	0.58	0.90	1.23	0.79	0.60	0.94	1.41	0.30	0.57	0.57	1.89	1.42	0.38	0.32	0.45	0.38
**10**	1.22	1.82	1.94	0.34	0.40	1.34	1.08	0.80	0.74	0.57	0.55	0.56	0.53	0.53	2.43	1.09	0.21	1.04	0.56	0.35
**11**	1.25	2.48	1.66	0.50	0.49	0.82	1.09	0.97	0.92	0.80	1.43	0.48	0.62	0.62	1.44	0.91	0.47	0.16	0.43	0.41
**12**	1.42	2.18	1.60	0.89	0.41	0.98	1.29	0.85	0.94	0.73	0.33	0.80	0.77	0.77	1.96	0.74	0.42	0.60	0.55	0.34
**13**	1.18	2.37	2.33	0.30	0.30	1.16	1.09	1.03	1.28	1.29	0.51	1.25	1.42	1.42	1.46	0.93	0.41	0.68	0.38	0.45
**14**	1.58	1.70	1.77	0.35	0.46	1.03	1.03	0.93	1.14	0.67	0.94	0.50	0.90	0.90	1.71	1.16	0.27	0.70	0.76	0.27
**15**	1.42	2.56	1.84	0.40	0.31	1.43	1.36	1.07	0.87	0.24	0.72	0.55	1.25	1.25	1.19	0.85	0.38	0.47	0.61	0.46

**16**	1.18	1.50	1.49	0.31	0.32	1.39	1.23	0.75	0.48	0.79	1.13	0.66	0.92	0.92	1.56	1.12	0.50	0.88	0.43	0.43
**17**	1.41	1.74	1.77	0.36	0.45	0.79	1.77	0.90	0.34	1.31	0.87	1.31	0.74	0.74	0.76	0.83	0.28	0.62	0.32	0.51
**18**	1.35	2.32	2.17	0.27	0.33	1.08	1.01	0.51	0.85	1.31	0.94	0.80	0.98	0.98	1.44	1.11	0.67	0.29	0.33	0.37
**19**	1.50	2.46	2.23	0.26	0.38	1.72	1.42	1.14	1.53	0.95	0.41	1.16	0.58	0.58	1.22	1.11	0.73	0.38	0.54	0.60
**20**	1.29	2.60	1.96	0.73	0.42	1.38	1.12	0.81	0.73	1.51	1.01	0.92	1.11	1.11	1.58	1.51	0.21	1.02	0.60	0.37
**21**	1.20	2.41	1.38	0.92	0.29	1.56	1.33	0.98	1.23	1.26	0.84	1.27	0.68	0.68	0.66	1.50	0.35	0.80	0.71	0.49
**22**	1.55	2.55	1.33	0.31	0.19	1.38	1.09	0.80	1.19	1.13	1.21	0.20	1.08	1.08	1.58	0.85	0.72	0.84	0.42	0.49
**23**	1.21	1.79	1.86	0.37	0.40	1.19	0.90	0.85	0.73	0.91	0.79	0.66	0.84	0.84	1.52	0.96	0.23	0.19	0.35	0.43
**24**	1.06	2.52	1.70	0.45	0.28	0.97	1.18	1.02	1.01	0.97	0.65	0.80	1.13	1.13	1.99	1.21	0.42	0.41	0.24	0.49
**25**	1.33	2.45	2.05	0.45	0.28	1.11	1.30	0.72	1.10	0.82	0.93	0.33	0.97	0.97	1.12	1.23	0.67	0.27	0.08	0.56
**26**	1.11	2.12	2.65	0.37	0.25	1.00	0.82	0.80	0.59	0.72	0.92	0.43	0.98	0.98	1.66	1.25	0.66	0.72	0.57	0.54
**27**	1.17	1.74	1.84	0.18	0.35	1.47	1.42	0.65	0.64	1.23	0.56	0.61	0.81	0.81	2.63	0.82	0.66	0.80	0.70	0.37
**28**	1.31	2.05	1.32	0.32	0.42	1.39	1.09	0.77	0.58	0.89	0.90	0.61	1.17	1.17	1.39	1.26	0.65	0.44	0.47	0.47
**29**	1.21	2.20	2.15	0.31	0.61	1.17	1.14	1.16	0.72	1.11	1.36	0.70	0.86	0.86	1.98	0.95	0.44	0.06	0.63	0.55
**30**	1.21	1.91	1.35	0.15	0.41	0.93	1.15	0.87	0.70	0.56	0.86	1.16	0.71	0.71	1.90	1.12	0.80	0.20	0.62	0.37
**31**	1.39	2.03	1.39	0.27	0.42	0.68	1.08	1.00	1.07	1.06	0.61	1.05	0.69	0.69	1.13	1.19	0.41	0.71	0.54	0.32
**32**	1.37	1.34	2.45	0.20	0.32	1.23	0.92	0.88	0.62	0.66	0.64	0.40	1.04	1.04	1.26	0.98	0.47	0.75	0.43	0.59
**33**	1.30	1.34	2.09	0.23	0.50	1.13	0.96	0.98	0.84	0.32	1.06	1.09	0.83	0.83	1.38	1.00	0.32	0.77	0.50	0.44
**34**	1.43	2.14	1.34	0.25	0.52	0.69	0.76	0.64	1.09	0.93	1.22	0.41	1.28	1.28	1.38	1.48	0.41	0.73	0.17	0.35
**35**	1.64	2.50	1.78	0.53	0.34	1.82	1.35	0.77	0.51	0.57	0.42	0.40	1.25	1.25	1.17	1.38	0.56	0.92	0.45	0.56
**36**	1.39	2.02	2.14	0.21	0.43	1.43	1.52	0.78	0.96	0.94	0.32	0.33	0.96	0.96	1.36	1.00	0.51	0.92	0.52	0.47
**37**	1.36	2.88	1.81	0.14	0.47	1.10	1.60	0.65	0.60	0.88	0.63	0.74	0.79	0.79	1.64	1.30	0.40	0.75	0.55	0.66
**38**	1.41	2.56	1.75	0.25	0.57	1.26	1.29	0.70	0.41	1.41	0.43	0.70	1.03	1.03	1.50	1.42	0.55	0.67	0.77	0.43
**39**	1.50	1.33	1.77	0.40	0.17	0.72	1.17	0.87	0.54	0.22	0.69	1.09	1.03	1.03	0.83	1.41	0.55	1.27	0.48	0.48
**40**	1.54	2.86	2.15	0.39	0.54	0.89	0.73	1.09	0.85	0.78	0.34	0.89	0.98	0.98	1.40	1.81	0.55	0.63	0.70	0.60

**41**	0.76	1.78	1.64	0.52	0.42	1.12	1.04	0.71	1.06	0.90	0.96	0.44	0.80	0.80	1.90	1.04	0.22	0.58	0.55	0.49
**42**	1.05	1.85	1.56	0.39	0.41	1.25	0.83	0.71	0.57	1.27	0.89	0.18	0.69	0.69	1.27	1.02	0.56	1.03	0.65	0.35
**43**	1.23	1.74	1.62	0.39	0.50	1.10	1.27	0.73	0.52	1.03	0.81	0.36	0.91	0.91	1.05	1.48	0.14	0.77	0.46	0.30
**44**	1.40	3.17	2.29	0.40	0.11	1.39	1.39	0.78	0.57	1.21	0.73	0.59	0.93	0.93	1.59	1.04	0.50	0.51	0.66	0.29
**45**	1.41	1.53	1.74	0.54	0.38	1.11	1.53	0.69	1.31	0.96	1.07	0.46	0.83	0.83	0.82	1.51	0.46	0.69	0.77	0.28
**46**	1.00	2.19	2.15	0.21	0.76	1.44	0.79	0.95	1.51	0.43	0.51	0.96	0.82	0.82	0.89	1.85	0.85	0.74	0.49	0.44
**47**	1.36	2.73	0.91	0.26	0.61	1.00	0.94	0.58	0.76	0.82	1.16	0.71	1.09	1.09	1.82	1.43	0.46	0.64	0.27	0.46
**48**	1.40	1.92	2.15	0.29	0.52	0.97	1.19	0.70	1.00	0.92	0.29	0.84	0.90	0.90	1.77	1.49	0.31	0.67	0.49	0.22
**49**	1.54	1.68	1.79	0.28	0.30	1.35	1.58	0.72	0.65	1.25	0.80	0.70	0.60	0.60	2.42	1.50	0.51	0.47	0.34	0.49
**50**	1.39	2.11	1.01	0.31	0.26	1.33	1.30	0.50	0.69	0.72	1.13	0.72	0.63	0.63	0.83	1.37	0.81	0.57	0.70	0.51
**51**	1.32	1.97	2.44	0.26	0.45	1.38	1.17	0.89	0.91	0.97	1.01	1.02	0.86	0.86	2.14	1.20	0.52	0.38	0.64	0.71
**52**	1.17	1.33	1.20	0.24	0.31	1.44	1.40	0.90	0.46	0.74	0.42	1.16	0.79	0.79	2.23	1.15	0.39	0.35	0.69	0.48
**53**	1.43	2.29	2.22	0.40	0.40	1.20	1.07	0.76	0.91	0.90	1.03	1.24	0.59	0.59	1.81	1.44	0.41	0.06	0.63	0.65
**54**	1.54	2.75	1.69	0.48	0.39	0.94	1.18	0.80	1.10	1.13	0.60	0.62	0.94	0.94	1.54	1.19	0.49	0.61	0.53	0.34
**55**	1.74	2.12	1.96	0.19	0.41	1.00	1.41	0.77	0.67	1.05	0.68	0.46	0.81	0.81	2.10	1.43	0.21	0.53	0.63	0.34
**56**	1.25	2.95	1.01	0.68	0.19	0.99	1.04	0.78	0.53	0.69	1.20	1.29	1.22	1.22	0.36	0.90	0.70	0.78	0.36	0.25
**57**	1.35	2.41	1.27	0.22	0.20	0.98	1.21	0.65	0.96	0.74	0.98	0.57	0.94	0.94	1.45	1.03	0.52	0.42	0.40	0.40
**58**	1.61	1.60	2.61	0.11	0.34	0.89	1.69	0.82	0.88	0.95	1.37	0.90	0.70	0.70	0.89	1.03	0.31	0.72	0.63	0.40
**59**	1.24	2.98	2.22	0.24	0.46	0.78	1.33	0.84	1.03	1.14	0.70	0.32	0.69	0.69	2.09	1.52	0.58	0.55	0.99	0.24
**60**	1.18	1.79	2.09	0.18	0.31	1.26	1.58	0.77	0.85	1.21	0.89	0.80	1.14	1.14	1.78	1.38	0.20	0.55	0.44	0.56
**61**	1.40	1.36	2.20	0.29	0.49	0.99	1.10	0.74	0.89	0.81	1.22	1.69	0.85	0.85	1.23	1.19	0.24	0.47	0.43	0.43
**62**	1.38	2.23	2.01	0.41	0.50	1.40	1.49	0.79	0.55	1.04	1.01	0.53	0.57	0.57	1.60	1.09	0.22	0.97	0.25	0.57
**63**	1.04	1.82	1.94	0.36	0.23	1.37	1.46	0.65	0.71	1.08	0.75	0.26	0.80	0.80	2.15	0.94	0.51	0.70	0.45	0.22
**64**	1.55	2.28	1.99	0.31	0.44	1.10	1.45	0.85	0.65	0.27	0.17	0.22	0.94	0.94	2.08	1.56	0.34	0.76	0.16	0.48
**65**	1.00	1.49	1.83	0.25	0.22	0.86	0.99	0.79	0.75	0.56	0.52	0.30	0.86	0.86	2.09	1.29	0.41	0.63	0.64	0.27

**66**	1.12	2.53	1.54	0.37	0.55	1.20	1.17	0.87	0.32	0.12	0.85	0.63	1.06	1.06	2.27	1.44	0.22	0.36	0.28	0.30
**67**	1.58	1.84	1.68	0.20	0.48	0.97	1.56	0.79	0.70	1.03	1.08	1.13	0.86	0.86	1.39	1.13	0.40	0.68	0.59	0.36
**68**	1.46	2.84	1.99	0.28	0.44	1.30	1.21	0.67	1.49	1.07	0.93	0.43	1.17	1.17	0.79	0.53	0.31	0.74	0.38	0.45
**69**	1.35	1.71	2.04	0.33	0.29	1.05	1.78	0.58	0.90	0.63	0.35	0.23	0.98	0.98	1.15	1.07	0.50	0.40	0.59	0.29
**70**	1.03	1.76	1.63	0.25	0.33	1.81	1.08	0.49	0.80	1.21	0.89	0.93	0.84	0.84	1.62	1.20	0.40	0.36	0.53	0.44
Mean value	1.33	2.10	1.84	0.35	0.38	1.15	1.20	0.79	0.83	0.88	0.82	0.67	0.90	0.90	1.54	1.19	0.45	0.62	0.51	0.43
