# Supplementary material for: Antibodies to synthetic citrullinated peptide epitope correlate with disease activity and flares in rheumatoid arthritis
Source: PLoS One. 2020 Apr 23;15(4):e0232010. doi: 10.1371/journal.pone.0232010 (PMC7179858; doi:10.1371/journal.pone.0232010)
Supplement: S3 Appendix — S3 Table. Onset data for RA subjects, longitudinal cohort (N = 30). S4 Table. Data for RA subjects 60 months after treatment. (PDF) [file pone.0232010.s003.pdf]

# S3 Appendix. RA cohort information

**S3 Table. Onset data for RA subjects, longitudinal cohort (N=30).**

| | | | | | | | | Serology | | | | | | Treatment | | |
|---|---|---|---|---|---|---|---|---|---|---|---|---|---|---|---|---|
| pat no | Gender | Age samp | Das28 at s | CDAI | SDAI | Caucasiar | Asian | RF | ACPA | ANA | MMP3 | ESR, mm/ | C4, mg/dl | NSAID | steroids | other* |
| 1 | F | 23 | 5 | 17 | 26 | Y | N | 1,50 | 1,40 | neg | neg | 7,40 | 27,00 | Y | Y | N |
| 2 | F | 32 | 5 | 18 | 28 | Y | N | 0,75 | 1,30 | neg | neg | 8,80 | 29,00 | Y | Y | N |
| 3 | F | 28 | 3 | 4 | 16 | Y | N | 0,91 | 1,40 | neg | neg | 9,20 | 17,00 | N | Y | N |
| 4 | F | 29 | 4 | 11 | 19 | Y | N | 1,40 | 1,20 | pos | neg | 11,30 | 18,00 | Y | Y | N |
| 5 | F | 40 | 4 | 25 | 20 | Y | N | 1,50 | 1,10 | neg | neg | 3,25 | 19,00 | Y | Y | Y |
| 6 | F | 28 | 5 | 20 | 22 | Y | N | 0,16 | 1,20 | neg | neg | 5,54 | 16,00 | N | N | N |
| 7 | F | 33 | 5 | 20 | 20 | Y | N | 0,49 | 0,95 | neg | neg | 7,60 | 21,00 | Y | Y | N |
| 8 | F | 35 | 4 | 19 | 21 | Y | N | 1,40 | 1,30 | neg | neg | 5,70 | 10,00 | Y | Y | N |
| 9 | F | 23 | 5 | 23 | 27 | Y | N | 2,10 | 0,97 | neg | neg | 8,20 | 10,00 | Y | Y | N |
| 10 | F | 27 | 7 | 40 | 36 | Y | N | 0,69 | 1,40 | pos | pos | 2,20 | 17,00 | N | Y | Y |
| 11 | F | 24 | 6 | 34 | 30 | Y | N | 1,00 | 0,98 | neg | neg | 9,89 | 11,00 | N | N | Y |
| 12 | F | 22 | 4 | 15 | 20 | Y | N | 0,97 | 1,10 | neg | neg | 5,6 | 18,30 | N | N | N |
| 13 | F | 24 | 5 | 22 | 26 | Y | N | 1,40 | 1,30 | neg | neg | 7,56 | 19,9 | Y | Y | N |
| 14 | F | 25 | 4 | 20 | 21 | Y | N | 0,80 | 1,20 | neg | neg | 4,43 | 28,00 | N | N | N |
| 15 | F | 30 | 5 | 30 | 26 | Y | N | 1,30 | 1,20 | neg | neg | 6,59 | 11,00 | N | Y | Y |
| 16 | F | 36 | 3 | 23 | 18 | N | Y | 1,30 | 0,99 | pos | pos | 2,80 | 19,00 | N | Y | N |
| 17 | F | 38 | 5 | 33 | 17 | Y | N | 0,93 | 1,50 | neg | neg | 4,34 | 22,00 | N | N | N |
| 18 | F | 32 | 5 | 36 | 20 | Y | N | 0,78 | 1,30 | neg | neg | 3,33 | 17,00 | Y | Y | N |
| 19 | F | 20 | 4 | 27 | 21 | Y | N | 1,00 | 1,40 | neg | neg | 9,20 | 11,00 | Y | Y | N |
| 20 | F | 33 | 7 | 43 | 35 | Y | N | 1,10 | 0,95 | pos | pos | 3,10 | 16,80 | N | N | Y |
| 21 | M | 28 | 5 | 36 | 22 | Y | N | 0,60 | 1,20 | neg | neg | 10,40 | 16,00 | Y | Y | N |
| 22 | F | 35 | 5 | 18 | 20 | Y | N | 1,70 | 1,10 | neg | neg | 10,20 | 9,90 | N | N | N |
| 23 | F | 22 | 5 | 14 | 17 | Y | N | 0,88 | 1,40 | neg | neg | 7,6 | 22,00 | N | Y | Y |
| 24 | M | 26 | 4 | 14 | 18 | Y | N | 1,20 | 1,50 | neg | neg | 6,57 | 20,00 | Y | Y | Y |
| 25 | M | 30 | 5 | 23 | 25 | Y | N | 0,65 | 0,99 | pos | neg | 4,32 | 18,00 | N | Y | N |
| 26 | F | 25 | 8 | 53 | 59 | N | Y | 1,30 | 1,50 | neg | pos | 2,11 | 14,00 | Y | Y | Y |
| 27 | M | 29 | 4 | 14 | 27 | Y | N | 0,89 | 1,50 | neg | neg | 5,71 | 23,00 | Y | Y | N |
| 28 | F | 31 | 5 | 21 | 22 | N | Y | 2,30 | 1,10 | neg | neg | 6,11 | 11,00 | Y | Y | Y |
| 29 | M | 30 | 2 | 1 | 2 | Y | N | 0,61 | 0,82 | neg | neg | 5,30 | 12,00 | Y | Y | N |
| 30 | F | 42 | 6 | 34 | 44 | Y | N | 0,86 | 1,10 | pos | pos | 3,00 | 10,00 | Y | Y | Y |

**S4 Table. Data for RA subjects 60 months after treatment.**

| pat no | Das28 at sample | CDAI | SDAI | RF | ACPA | ANA | MMP3 | ESR | C4 | NSAID | steroids | other* |
|---|---|---|---|---|---|---|---|---|---|---|---|---|
| 1 | 4 | 12 | 11 | 0,21 | 0,61 | neg | neg | 11,00 | 9,00 | N | N | N |
| 2 | 6 | 17 | 21 | 0,21 | 1,20 | neg | neg | 9,11 | 8,00 | Y | N | N |
| 3 | 5 | 13 | 12 | 0,22 | 2,20 | neg | neg | 10,20 | 13,00 | N | Y | N |
| 4 | 6 | 17 | 21 | 0,08 | 2,20 | pos | neg | 11,30 | 8,00 | Y | Y | N |

| | | | | | | | | | | | | |
|---|---|---|---|---|---|---|---|---|---|---|---|---|
| 5 | 5 | 14 | 14 | 0,18 | 2,00 | neg | neg | 5,43 | 8,00 | Y | Y | N |
| 6 | 4 | 12 | 11 | 0,16 | 2,10 | neg | neg | 5,54 | 15,00 | N | N | N |
| 7 | 5 | 16 | 19 | 0,22 | 2,10 | neg | neg | 8,82 | 17,00 | Y | Y | N |
| 8 | 2 | 6 | 5 | 0,14 | 1,80 | neg | neg | 9,12 | 13,40 | Y | Y | N |
| 9 | 4 | 12 | 10 | 0,12 | 1,50 | neg | neg | 32,00 | 15,00 | Y | N | N |
| 10 | 3 | 9 | 6 | 0,24 | 1,40 | pos | neg | 16,90 | 11,00 | N | Y | Y |
| 11 | 8 | 30 | 22 | 0,14 | 2,60 | neg | neg | 10,54 | 12,00 | N | N | N |
| 12 | 5 | 14 | 13 | 0,18 | 3,10 | neg | neg | 12,23 | 10,00 | N | N | N |
| 13 | 6 | 18 | 21 | 0,19 | 2,11 | neg | neg | 9,91 | 14 | N | Y | N |
| 14 | 6 | 18 | 16 | 0,22 | 0,61 | neg | neg | 11,20 | 11,00 | N | N | N |
| 15 | 7 | 20 | 19 | 0,35 | 1,20 | neg | neg | 6,59 | 8,80 | N | Y | Y |
| 16 | 6 | 16 | 17 | 0,21 | 3,00 | pos | pos | 11,20 | 9,00 | N | N | N |
| 17 | 4 | 12 | 11 | 0,27 | 1,70 | neg | neg | 4,34 | 21,00 | N | N | N |
| 18 | 6 | 16 | 15 | 0,24 | 2,20 | neg | neg | 10,14 | 12,00 | N | N | N |
| 19 | 6 | 17 | 19 | 0,23 | 1,50 | neg | neg | 11,20 | 14,00 | Y | N | N |
| 20 | 6 | 18 | 18 | 0,16 | 2,00 | pos | pos | 24,00 | 9,00 | N | N | Y |
| 21 | 4 | 12 | 11 | 0,21 | 1,60 | neg | neg | 10,40 | 17,00 | Y | Y | N |
| 22 | 7 | 20 | 19 | 0,24 | 1,11 | neg | neg | 11,22 | 17,00 | N | N | N |
| 23 | 8 | 26 | 26 | 0,15 | 0,35 | neg | neg | 7,6 | 21,00 | N | Y | Y |
| 24 | 7 | 24 | 19 | 0,12 | 1,60 | neg | neg | 6,57 | 5,00 | N | N | Y |
| 25 | 8 | 22 | 22 | 0,26 | 1,70 | pos | neg | 4,41 | 16,00 | N | Y | N |
| 26 | 6 | 16 | 16 | 0,22 | 2,20 | neg | pos | 5,54 | 17,00 | Y | Y | Y |
| 27 | 6 | 17 | 17 | 0,24 | 1,50 | neg | neg | 20,00 | 11,00 | Y | Y | N |
| 28 | 7 | 19 | 18 | 0,25 | 1,45 | neg | neg | 6,11 | 12,00 | Y | Y | Y |
| 29 | 4 | 12 | 13 | 0,12 | 1,60 | neg | neg | 5,30 | 13,20 | Y | Y | N |
| 30 | 2 | 6 | 5 | 0,11 | 2,00 | pos | neg | 3,00 | 10,00 | Y | Y | Y |

Cut off for positivity, 1.5, RF; 1.4, ACPA. ANA has been done by HEP2 assay at clinical lab. Treatment, others* - using biological drugs.
