# Supplementary material for: Antibodies to synthetic citrullinated peptide epitope correlate with disease activity and flares in rheumatoid arthritis
Source: PLoS One. 2020 Apr 23;15(4):e0232010. doi: 10.1371/journal.pone.0232010 (PMC7179858; doi:10.1371/journal.pone.0232010)
Supplement: S4 Appendix — S5 Table. Data for longitudinal SLE subjects at onset, N = 30. S6 Table. Data for SLE subjects 60 months after treatment. (PDF) [file pone.0232010.s004.pdf]

S4 Appendix. SLE cohort information

S5 Table. Data for longitudinal SLE subjects at onset, N = 30.

**SLE cohort - onset**						Serology						Treatment		
pat no	Gender	Age samp	SLEDAI at sample	Caucasiar	Asian	RF	ANA	ACPA	MMP3	ESR, mm/	C4, mg/dl	NSAID	steroids	other*
1	F	16	5	Y	N	0,07	2,20	0,47	neg	9,09	15,00	N	Y	N
2	M	33	6	Y	N	0,07	1,30	0,39	neg	7,53	21,00	N	Y	N
3	F	20	1	Y	N	0,06	1,90	0,63	neg	8,43	28,00	Y	N	N
4	M	21	10	N	Y	0,06	1,60	0,49	neg	9,34	21,00	Y	Y	Y
5	F	25	3	Y	N	0,06	2,40	0,39	neg	5,51	15,00	Y	Y	N
6	F	29	0	Y	N	0,06	1,50	0,43	neg	8,89	21,00	Y	N	N
7	M	30	5	Y	N	0,07	1,70	0,44	neg	7,29	23,00	N	N	N
8	M	32	11	Y	N	0,05	1,40	0,42	pos	7,54	25,00	Y	Y	Y
9	F	33	0	Y	N	0,06	1,20	0,51	neg	6,80	31,00	N	N	N
10	F	21	9	Y	N	0,06	1,90	0,39	neg	2,44	32,00	Y	Y	Y
11	F	20	10	N	Y	0,06	1,80	0,49	neg	1,40	30,00	Y	Y	Y
12	F	19	5	Y	N	0,07	1,50	0,59	neg	9,32	24,56	N	Y	N
13	F	17	7	Y	N	0,06	2,20	0,48	neg	8,19	11,00	N	N	N
14	F	26	5	Y	N	0,05	0,98	0,62	neg	9,26	9,00	Y	Y	N
15	F	22	5	Y	N	0,06	1,50	0,45	neg	5,45	10,00	Y	Y	N
16	M	34	2	Y	N	0,07	1,00	0,50	neg	2,60	20,00	N	N	Y
17	F	38	6	Y	N	0,07	2,00	0,45	neg	3,59	22,00	N	N	N
18	F	30	4	Y	N	0,06	2,50	0,68	neg	8,38	24,00	Y	N	N
19	F	21	8	Y	N	0,07	1,20	0,57	neg	9,26	28,00	Y	Y	Y
20	M	22	7	Y	N	0,07	1,40	0,56	neg	9,30	20,00	Y	N	N
21	M	25	6	Y	N	0,06	2,80	0,51	neg	8,60	21,00	Y	N	N
22	F	35	6	Y	N	0,06	1,80	0,66	neg	9,27	18,00	Y	Y	N
23	F	20	6	Y	N	0,07	2,20	0,60	neg	6,28	15,00	Y	Y	Y
24	F	17	7	Y	N	0,06	1,40	0,64	neg	5,43	16,20	Y	Y	Y
25	F	21	7	Y	N	0,06	1,30	0,52	neg	3,64	19,30	Y	Y	N
26	F	23	2	Y	N	0,06	0,56	0,58	neg	8,73	22,89	N	N	N
27	F	22	10	N	Y	0,07	0,99	0,35	neg	2,65	12,43	Y	Y	Y
28	F	34	11	Y	N	0,06	2,10	0,71	neg	5,05	23,00	Y	Y	Y
29	F	29	6	Y	N	0,05	2,10	0,57	neg	4,38	30,00	Y	N	N
30	F	35	7	Y	N	0,06	1,20	0,38	neg	2,48	20,28	Y	N	N

S6 Table. Data for SLE subjects 60 months after treatment.

pat no	SLEDAI	RF	ANA	ACPA	MMP3	ESR	C4	NSAID	steroids	other*
1	4	0,16	1,70	0,37	neg	3,09	10,70	N	N	N
2	6	0,14	1,40	0,30	neg	1,53	16,70	N	N	N
3	5	0,19	1,10	0,24	neg	2,43	23,70	N	N	N
4	6	0,21	0,56	0,31	neg	3,34	21,00	N	Y	N
5	5	0,27	1,00	0,28	neg	2,40	11,00	Y	N	N
6	4	0,18	0,97	0,37	neg	2,89	13,00	Y	N	N
7	5	0,32	1,20	0,28	neg	1,29	14,70	N	N	N

8	2	0,44	1,30	0,30	pos	1,54	20,70	N	Y	N
9	4	0,33	1,30	0,28	neg	0,80	26,70	N	N	N
10	3	0,32	0,67	0,23	neg	3,80	27,70	Y	Y	Y
11	8	0,17	1,50	0,27	neg	2,40	15,00	N	N	Y
12	5	0,31	0,96	0,39	neg	3,32	11,00	N	Y	N
13	6	0,44	0,50	0,42	neg	2,19	16,00	N	N	N
14	6	0,23	1,30	0,19	neg	3,26	4,70	Y	Y	N
15	7	0,10	1,10	0,33	neg	5,55	5,70	Y	N	N
16	6	0,19	0,61	0,28	neg	2,40	15,70	N	N	Y
17	4	0,16	0,89	0,35	neg	1,40	17,70	N	N	N
18	6	0,24	0,84	0,33	neg	2,38	21,00	Y	N	N
19	6	0,15	0,99	0,25	neg	3,26	23,70	Y	N	N
20	6	0,31	1,60	0,31	neg	3,30	15,70	N	N	N
21	4	0,18	1,80	0,22	neg	2,60	16,70	Y	N	N
22	7	0,24	1,70	0,27	neg	3,27	13,70	Y	N	N
23	8	0,11	1,50	0,21	neg	0,28	10,70	Y	N	N
24	7	0,19	1,40	0,42	neg	0,57	11,90	Y	Y	Y
25	8	0,45	2,70	0,30	neg	4,30	9,00	Y	Y	N
26	6	0,17	1,50	0,20	neg	2,73	18,59	N	N	N
27	6	0,04	1,60	0,13	neg	0,33	8,13	Y	Y	N
28	7	0,32	1,70	0,29	neg	0,95	18,70	Y	N	N
29	4	0,27	1,10	0,22	neg	1,62	25,70	N	N	N
30	2	0,38	1,40	0,32	neg	3,52	15,98	Y	N	N

Cut off for positivity, 1.5, RF; 1.4, ACPA. ANA has been done by HEP2 assay at clinical lab. Treatment, others* - using biological drugs.
