# Supplementary material for: Antibodies to synthetic citrullinated peptide epitope correlate with disease activity and flares in rheumatoid arthritis
Source: PLoS One. 2020 Apr 23;15(4):e0232010. doi: 10.1371/journal.pone.0232010 (PMC7179858; doi:10.1371/journal.pone.0232010)
Supplement: S5 Appendix — (PDF) [file pone.0232010.s005.pdf]

S5 Appendix. Healthy controls - information

S7 Table. Information on healthy controls used in the longitudinal study.

					Serology						Treatment
Subject nc	Gender	Age samp	Caucasiar	Asian	RF	ANA	ACPA	MMP3	ESR, mm/	C4, mg/dl	incl. Supplements
1	F	22	Y	N	0,07	0,27	0,28	neg	5,70	25,00	N
2	M	40	N	Y	0,07	0,15	0,44	neg	4,30	41,00	N
3	F	21	Y	N	0,06	0,21	0,35	neg	5,00	28,00	N
4	M	27	Y	N	0,06	0,16	0,39	neg	4,10	32,00	Y
5	F	33	Y	N	0,06	0,28	0,45	neg	4,10	35,00	N
6	F	42	Y	N	0,08	0,19	0,32	neg	4,10	30,00	N
7	F	44	Y	N	0,11	0,37	0,31	neg	4,90	26,00	N
8	F	30	Y	N	0,25	0,17	0,35	neg	5,20	42,00	N
9	F	32	Y	N	0,07	0,26	0,40	neg	6,80	31,00	N
10	F	25	Y	N	0,06	0,28	0,23	neg	4,60	32,00	N
11	F	20	Y	N	0,06	0,22	0,33	neg	4,80	30,00	N
12	F	20	Y	N	1,50	1,12	0,38	neg	3,80	24,56	Y
13	F	31	Y	N	0,11	0,14	0,31	neg	3,80	27,00	N
14	M	35	Y	N	0,24	0,33	0,27	neg	4,40	26,00	N
15	M	32	Y	N	0,07	0,28	0,27	neg	3,70	34,00	N
16	F	44	N	Y	0,05	0,40	0,34	neg	5,20	30,00	N
17	M	40	Y	N	0,04	0,38	0,16	neg	4,60	29,00	N
18	M	35	Y	N	0,11	0,14	0,32	neg	3,70	25,00	N
19	F	33	Y	N	0,12	0,28	0,35	neg	4,60	28,00	N
20	F	30	Y	N	0,22	0,22	0,42	neg	3,90	24,00	N
21	F	29	Y	N	0,43	0,16	0,36	neg	5,10	31,00	N
22	F	26	Y	N	0,54	0,16	0,28	neg	4,20	38,00	N
23	F	22	Y	N	0,12	0,19	0,34	neg	4,80	25,00	N
24	F	26	Y	N	0,07	0,39	0,40	neg	3,10	26,00	N
25	F	30	Y	N	0,11	0,29	0,33	neg	4,50	29,00	N
26	F	25	Y	N	0,14	0,27	0,24	neg	5,40	22,89	N
27	F	29	Y	N	0,07	0,16	0,25	neg	4,00	42,00	N
28	F	21	Y	N	0,06	0,34	0,23	neg	3,00	32,00	Y
29	F	30	Y	N	0,05	0,20	0,32	neg	4,40	40,00	N
30	F	42	N	Y	0,06	0,27	0,31	neg	3,20	30,00	N
