# Supplementary material for: Antibodies to synthetic citrullinated peptide epitope correlate with disease activity and flares in rheumatoid arthritis
Source: PLoS One. 2020 Apr 23;15(4):e0232010. doi: 10.1371/journal.pone.0232010 (PMC7179858; doi:10.1371/journal.pone.0232010)
Supplement: S6 Appendix — S8 Table. Mean A450 values, RA cohort (N = 30). S9 Table. Data for longitudinal study of RA cohort (N = 30). (PDF) [file pone.0232010.s006.pdf]

S6 Appendix. Longitudinal study, RA cohort, individual data points.

S8 Table. Mean A450 values, RA cohort (N = 30)

time	mean sco	E1	E2	E3	RF	ANA	ACPA
0	5	0,1	0,09	0,13	1,1	0,08	1,32
3	3	0,21	0,23	0,07	2,11	0,11	1,1
6	4	1,9	2,9	0,87	1,8	0,25	2,11
9	3	0,11	0,43	0,67	0,78	0,13	1,2
12	2	0,06	1,2	0,1	0,67	0,35	1,23
15	4	0,22	1,66	0,08	0,56	0,22	1,22
18	8	1,1	2,1	0,07	0,76	0,22	1,4
21	5	1,86	0,25	0,13	0,45	0,35	1,7
24	6	0,12	0,13	0,33	0,55	0,13	1,19
27	6	0,23	0,34	0,32	0,45	0,24	1,7
30	5	0,19	0,21	0,04	0,45	0,07	1,1
33	4	1,22	1,21	0,33	0,31	0,31	1
36	6	0,11	0,34	0,32	0,45	0,45	1,2
39	4	0,07	2,11	0,05	0,27	0,07	1,1
42	3	2,12	2,77	0,12	0,16	0,06	1,86
45	2	0,73	1,77	0,23	0,17	0,07	1,12
48	6	1,24	1,31	0,21	0,11	0,11	1,23
51	4	0,1	1,45	0,13	0,08	0,08	1,19
54	6	0,21	2,7	0,07	0,09	0,09	1,25
57	6	0,12	1,6	0,19	0,24	0,24	1,11
60	4	0,23	2,1	0,68	0,2	0,21	1,7

S9 Table. Data for longitudinal study of RA cohort (N = 30)

2	time point						
Pat no	0						
	score	E1	E2	E3	RF	ANA	ACPA
1	5	0,09	0,14	0,20	1,50	0,07	1,40
2	5	0,11	0,07	0,06	0,75	0,07	1,30
3	3	0,13	0,05	0,18	0,91	0,08	1,40
4	4	0,13	0,09	0,08	1,40	0,09	1,20
5	4	0,12	0,09	0,21	1,50	0,08	1,10
6	5	0,08	0,14	0,06	0,16	0,07	1,20
7	5	0,06	0,10	0,14	0,49	0,09	0,95
8	4	0,13	0,10	0,07	1,40	0,08	1,30
9	5	0,10	0,08	0,11	2,10	0,07	0,97
10	7	0,13	0,12	0,11	0,69	0,07	1,40
11	6	0,11	0,10	0,17	1,00	0,08	0,98
12	4	0,11	0,10	0,13	0,97	0,07	1,10
13	5	0,12	0,09	0,23	1,40	0,06	1,30
14	4	0,07	0,05	0,15	0,80	0,08	1,20
15	5	0,08	0,08	0,13	1,30	0,06	1,20
16	3	0,13	0,11	0,08	1,30	0,09	0,99
17	5	0,08	0,08	0,14	0,93	0,10	1,50
18	5	0,09	0,10	0,03	0,78	0,07	1,30
19	4	0,10	0,09	0,10	1,00	0,07	1,40
20	7	0,11	0,11	0,07	1,10	0,07	0,95
21	5	0,07	0,13	0,07	0,60	0,08	1,20
22	5	0,10	0,08	0,07	1,70	0,06	1,10
23	5	0,11	0,10	0,12	0,88	0,09	1,40
24	4	0,08	0,06	0,13	1,20	0,08	1,50
25	5	0,10	0,10	0,18	0,65	0,08	0,99
26	8	0,10	0,07	0,19	1,30	0,09	1,50
27	4	0,12	0,10	0,05	0,89	0,09	1,50
28	5	0,08	0,07	0,09	2,30	0,08	1,10
29	2	0,11	0,08	0,17	0,61	0,09	0,82
30	6	0,08	0,09	0,08	0,86	0,09	1,10

	time point						
Pat no	3						
	score	E1	E2	E3	RF	ANA	ACPA
1	2	0,25	0,23	0,06	2,50	0,07	1,20
2	4	0,20	0,23	0,06	2,40	0,10	0,89
3	3	0,23	0,22	0,05	3,30	0,07	1,20
4	3	0,18	0,22	0,07	2,70	0,09	1,20
5	3	0,21	0,23	0,06	2,60	0,09	1,20
6	4	0,23	0,22	0,07	2,20	0,08	1,20
7	3	0,27	0,24	0,06	2,90	0,09	1,00
8	2	0,20	0,22	0,07	2,90	0,07	1,20
9	1	0,14	0,24	0,06	2,20	0,07	1,10
10	4	0,11	0,24	0,07	2,20	0,09	1,10
11	3	0,13	0,21	0,07	2,20	0,09	1,10
12	3	0,19	0,24	0,08	2,00	0,08	1,20
13	2	0,20	0,24	0,08	1,70	0,07	1,10
14	4	0,24	0,22	0,08	1,90	0,07	0,98
15	2	0,14	0,23	0,07	2,10	0,08	1,10
16	4	0,25	0,23	0,09	1,70	0,08	1,00
17	3	0,19	0,25	0,08	2,70	0,08	1,10
18	4	0,22	0,23	0,07	1,40	0,09	1,00
19	5	0,17	0,24	0,07	2,70	0,07	1,10
20	3	0,11	0,21	0,06	2,10	0,08	1,10
21	3	0,21	0,24	0,09	2,00	0,08	1,10
22	5	0,20	0,23	0,09	2,70	0,09	1,10
23	5	0,15	0,24	0,07	3,50	0,09	1,10
24	2	0,17	0,24	0,06	2,00	0,09	1,10
25	3	0,20	0,24	0,07	1,60	0,08	1,00
26	3	0,17	0,21	0,08	1,80	0,09	1,20
27	4	0,26	0,23	0,07	2,10	0,07	0,96
28	4	0,25	0,23	0,05	0,25	0,06	1,40
29	4	0,19	0,21	0,07	2,50	0,08	0,98
30	2	0,31	0,23	0,07	2,60	0,07	1,10

	time point						
Pat no	6						
	score	E1	E2	E3	RF	ANA	ACPA
1	5	1,60	2,30	0,88	1,50	0,13	2,30
2	5	2,20	3,40	0,87	2,60	0,10	1,90
3	6	1,80	3,10	0,87	0,81	0,10	1,80
4	6	1,70	2,70	0,86	1,80	0,13	2,50
5	5	1,80	2,90	0,87	2,30	0,08	2,40
6	5	2,30	3,40	0,89	1,10	0,13	1,80
7	4	2,10	3,60	0,88	1,90	0,13	1,80
8	6	2,10	3,20	0,87	1,30	0,00	2,70
9	4	2,30	3,20	0,86	1,70	0,12	1,50
10	4	1,80	3,30	0,87	1,80	0,12	3,00
11	3	1,70	3,50	0,89	2,20	0,14	2,80
12	4	1,90	3,40	0,87	0,66	0,08	2,30
13	4	2,30	2,80	0,88	0,77	0,11	2,80
14	5	2,00	2,80	0,86	3,10	0,08	2,80
15	2	1,70	2,80	0,85	1,60	0,13	2,10
16	4	2,00	2,80	0,86	1,70	0,10	1,90
17	7	1,90	3,40	0,85	0,42	0,11	2,20
18	3	1,30	2,60	0,89	0,87	0,08	1,10
19	4	2,70	2,60	0,87	1,30	0,12	3,00
20	3	2,20	3,00	0,86	2,00	0,09	2,60
21	3	2,00	3,20	0,86	2,20	0,11	3,30
22	4	1,90	2,60	0,86	1,70	0,15	2,60
23	4	1,70	3,20	0,88	1,90	0,14	2,00
24	3	2,30	2,60	0,88	0,70	0,10	2,90
25	4	1,50	2,70	0,89	1,20	0,19	1,40
26	5	1,80	3,00	0,89	1,10	0,10	2,20
27	6	1,60	3,30	0,85	2,20	0,14	1,90
28	5	1,90	3,50	0,87	1,30	0,07	2,20
29	4	1,80	3,50	0,87	1,80	0,16	2,20
30	4	1,70	2,90	0,87	1,10	0,09	1,80

	time point						
Pat no	9						
	score	E1	E2	E3	RF	ANA	ACPA
1	3	0,11	0,46	0,66	1,20	0,26	0,94
2	1	0,12	0,35	0,66	1,00	0,28	1,30
3	3	0,12	0,33	0,67	0,66	0,30	1,30
4	2	0,10	0,38	0,69	0,83	0,26	0,85
5	3	0,12	0,40	0,66	0,58	0,20	1,10
6	3	0,11	0,44	0,67	0,86	0,20	1,60
7	2	0,16	0,43	0,70	0,92	0,22	1,20
8	1	0,11	0,58	0,66	0,88	0,27	1,30
9	2	0,12	0,38	0,67	0,42	0,24	1,20
10	4	0,09	0,62	0,66	0,68	0,25	1,10
11	3	0,11	0,31	0,67	0,77	0,23	0,94
12	3	0,07	0,51	0,68	0,59	0,23	1,40
13	3	0,12	0,37	0,67	0,60	0,27	1,10
14	4	0,08	0,42	0,67	1,10	0,17	1,30
15	4	0,10	0,47	0,68	0,93	0,30	1,40
16	2	0,07	0,41	0,68	1,20	0,30	1,70
17	2	0,10	0,43	0,68	0,73	0,21	0,77
18	4	0,16	0,33	0,67	0,48	0,19	1,70
19	3	0,12	0,43	0,68	1,00	0,27	1,20
20	3	0,12	0,46	0,66	0,67	0,20	1,40
21	2	0,16	0,41	0,68	1,10	0,23	1,10
22	2	0,11	0,55	0,66	0,55	0,29	1,00
23	1	0,09	0,39	0,69	0,82	0,28	0,92
24	3	0,07	0,38	0,69	0,50	0,21	1,70
25	3	0,10	0,38	0,68	1,10	0,28	1,30
26	5	0,13	0,41	0,69	0,52	0,23	0,91
27	3	0,11	0,49	0,67	0,56	0,31	1,50
28	3	0,07	0,41	0,68	0,53	0,19	1,10
29	4	0,12	0,28	0,67	0,86	0,29	1,10
30	1	0,17	0,43	0,66	0,57	0,21	1,10

	time point						
Pat no	12						
	score	E1	E2	E3	RF	ANA	ACPA
1	4	0,04	1,30	0,08	0,72	0,13	1,70
2	1	0,07	0,94	0,09	1,10	0,16	0,87
3	1	0,06	1,10	0,12	0,92	0,14	1,80
4	3	0,07	1,40	0,13	1,00	0,14	1,50
5	2	0,08	1,00	0,11	0,90	0,13	1,10
6	1	0,11	1,30	0,12	0,89	0,09	0,66
7	2	0,07	1,40	0,12	0,65	0,12	1,00
8	2	0,06	1,30	0,12	0,69	0,15	0,67
9	2	0,08	0,89	0,11	1,10	0,11	0,72
10	3	0,07	1,10	0,06	0,62	0,10	1,50
11	3	0,05	1,30	0,11	0,36	0,08	1,00
12	0	0,07	0,87	0,11	0,41	0,18	1,90
13	1	0,06	0,82	0,13	0,87	0,10	0,87
14	2	0,05	1,00	0,12	0,88	0,19	0,83
15	1	0,05	1,30	0,13	0,88	0,14	2,40
16	3	0,02	1,30	0,12	0,98	0,14	0,97
17	2	0,08	1,30	0,07	0,91	0,08	0,85
18	2	0,07	1,10	0,12	0,67	0,11	1,10

	time point						
Pat no	15						
	score	E1	E2	E3	RF	ANA	ACPA
1	4	0,19	1,50	0,06	0,60	0,30	1,00
2	3	0,27	1,50	0,10	0,78	0,31	0,47
3	3	0,23	1,70	0,11	0,63	0,34	1,40
4	4	0,22	1,80	0,13	0,31	0,32	0,74
5	4	0,27	1,80	0,13	0,70	0,31	1,30
6	5	0,21	1,80	0,06	0,62	0,42	0,22
7	4	0,21	1,70	0,17	0,60	0,35	1,40
8	5	0,25	1,50	0,14	0,82	0,31	1,20
9	5	0,20	2,00	0,12	0,39	0,43	2,00
10	5	0,25	1,80	0,09	0,59	0,29	1,60
11	5	0,31	1,60	0,10	0,69	0,33	1,10
12	4	0,23	1,40	0,08	0,18	0,38	0,96
13	5	0,20	1,40	0,05	0,64	0,47	0,60
14	3	0,22	1,40	0,14	0,63	0,34	0,74
15	4	0,16	1,80	0,14	0,49	0,38	1,30
16	3	0,15	1,40	0,07	0,79	0,38	0,82
17	3	0,23	1,50	0,08	0,65	0,27	1,40
18	6	0,18	1,60	0,09	0,66	0,29	1,60

	time point						
Pat no	18						
	score	E1	E2	E3	RF	ANA	ACPA
1	9	0,77	2,20	0,10	0,55	0,26	0,74
2	9	0,88	2,20	0,11	0,67	0,15	1,10
3	8	1,30	2,30	0,06	0,75	0,16	1,20
4	8	0,67	1,90	0,05	0,60	0,19	0,47
5	8	0,92	1,90	0,08	0,32	0,23	1,20
6	9	1,80	2,20	0,08	0,86	0,18	1,30
7	8	1,10	2,40	0,02	0,72	0,17	1,20
8	8	1,00	2,10	0,08	0,93	0,21	1,00
9	8	0,93	2,40	0,04	0,68	0,22	1,00
10	7	0,81	2,20	0,11	0,73	0,23	1,10
11	9	1,70	2,20	0,05	0,75	0,24	0,79
12	7	1,10	2,30	0,12	0,59	0,29	0,61
13	8	1,00	1,80	0,04	0,88	0,21	0,72
14	8	0,92	2,00	0,04	0,84	0,26	1,70
15	9	0,91	2,50	0,06	0,80	0,20	1,40
16	10	1,60	2,20	0,11	0,64	0,25	0,51
17	8	1,60	2,50	0,04	0,92	0,24	1,10
18	8	1,20	1,80	0,05	0,65	0,26	0,59

	time point						
Pat no	21						
	score	E1	E2	E3	RF	ANA	ACPA
1	6	1,80	0,27	0,08	0,31	0,09	0,98
2	5	1,60	0,32	0,10	0,25	0,22	1,10
3	6	2,20	0,37	0,09	0,68	0,32	2,20
4	5	1,40	0,19	0,05	0,44	0,32	1,70
5	3	2,40	0,31	0,03	0,70	0,34	1,90
6	6	1,30	0,54	0,03	0,40	0,10	1,30
7	5	1,90	0,19	0,06	0,14	0,32	1,20
8	6	1,50	0,33	0,09	0,36	0,23	0,65
9	6	1,90	0,23	0,04	0,50	0,14	1,00
10	5	1,80	0,04	0,08	0,19	0,27	0,83
11	4	1,50	0,10	0,06	0,22	0,36	1,40
12	4	1,60	0,10	0,11	0,60	0,15	1,00
13	6	2,00	0,37	0,08	0,75	0,27	2,10
14	6	2,10	0,37	0,08	0,53	0,22	1,30
15	5	2,50	0,40	0,12	0,61	0,21	1,60
16	6	1,90	0,08	0,04	0,39	0,10	0,87
17	6	1,40	0,20	0,06	0,72	0,18	0,98
18	7	1,70	0,27	0,04	0,62	0,29	0,95

19	1	0,04	0,93	0,14	0,81	0,10	1,20	3	0,20	1,90	0,08	0,47	0,33	0,89	8	0,77	2,00	0,08	1,30	0,27	0,62	4	2,20	0,11	0,07	0,21	0,22	0,94
20	1	0,10	1,20	0,07	0,66	0,17	1,70	2	0,26	1,70	0,08	0,85	0,29	1,20	8	0,73	1,90	0,05	0,52	0,17	1,70	5	1,40	0,21	0,06	0,35	0,23	1,90
21	2	0,09	1,70	0,09	0,71	0,14	0,89	3	0,20	1,40	0,05	0,62	0,32	1,10	8	1,30	1,70	0,08	0,60	0,24	1,10	3	1,60	0,27	0,05	0,32	0,27	0,94
22	2	0,06	1,30	0,12	0,97	0,12	0,90	5	0,23	1,60	0,09	0,74	0,46	1,40	7	1,40	1,60	0,12	0,66	0,24	0,90	7	1,50	0,17	0,05	0,21	0,16	1,90
23	4	0,03	1,00	0,11	0,65	0,16	1,70	4	0,10	1,60	0,13	0,85	0,34	1,80	7	0,72	2,20	0,15	0,97	0,36	0,91	6	1,90	0,20	0,03	0,47	0,33	1,60
24	2	0,09	1,10	0,08	0,57	0,14	1,20	3	0,20	1,80	0,08	0,70	0,37	0,53	9	0,99	1,40	0,04	1,00	0,24	0,43	5	2,40	0,26	0,11	0,47	0,30	2,10
25	3	0,04	0,88	0,12	0,46	0,12	0,56	4	0,29	1,60	0,14	0,48	0,36	2,00	8	0,76	2,10	0,10	0,79	0,21	1,00	5	1,90	0,17	0,09	0,26	0,14	0,23
26	1	0,06	0,92	0,11	0,77	0,06	1,40	5	0,22	1,90	0,11	0,68	0,36	0,70	9	1,30	1,70	0,05	0,60	0,21	0,97	5	1,30	0,21	0,02	0,95	0,21	1,00
27	1	0,08	1,30	0,09	0,65	0,17	1,60	3	0,21	1,30	0,16	0,87	0,41	0,48	8	0,51	2,20	0,08	0,81	0,24	1,60	6	1,70	0,05	0,11	0,09	0,23	1,10
28	1	0,07	1,20	0,10	0,82	0,09	0,92	4	0,22	1,50	0,13	0,34	0,29	1,20	8	1,40	1,90	0,09	0,39	0,30	1,00	5	0,65	0,45	0,10	0,39	0,20	1,30
29	2	0,06	1,10	0,13	0,41	0,17	0,95	5	0,20	1,60	0,02	0,60	0,37	0,96	9	0,96	2,40	0,11	1,10	0,13	1,20	5	1,80	0,28	0,05	0,30	0,14	0,68
30	3	0,05	1,10	0,11	0,62	0,14	1,80	5	0,25	1,90	0,09	0,84	0,36	1,40	8	1,70	2,30	0,03	0,52	0,21	1,10	4	1,90	0,24	0,07	0,35	0,23	1,50

Pat no	time point 24 score	E1	E2	E3	RF	ANA	ACPA	time point 27 score	E1	E2	E3	RF	ANA	ACPA	time point 30 score	E1	E2	E3	RF	ANA	ACPA	time point 33 score	E1	E2	E3	RF	ANA	ACPA
1	6	0,11	0,11	0,56	0,49	0,36	1,40	7	0,21	0,33	0,13	0,62	0,15	0,77	6	0,29	0,23	0,36	0,46	0,14	1,80	4	1,20	0,99	0,38	0,94	0,07	0,99
2	5	0,11	0,09	0,37	0,61	0,38	1,80	5	0,23	0,38	0,17	0,42	0,19	0,47	5	0,20	0,23	0,25	0,57	0,13	0,91	4	1,20	1,40	0,40	0,51	0,08	0,74
3	6	0,15	0,13	0,23	0,89	0,42	1,50	6	0,25	0,42	0,17	0,54	0,04	0,76	6	0,18	0,23	0,34	0,44	0,30	0,93	3	1,10	0,91	0,31	0,42	0,08	1,30
4	5	0,15	0,15	0,40	0,51	0,30	1,00	6	0,25	0,37	0,11	0,18	0,21	1,20	5	0,12	0,17	0,28	0,22	0,18	2,70	4	1,20	1,30	0,31	0,45	0,05	0,85
5	3	0,11	0,16	0,21	0,51	0,37	1,80	7	0,25	0,39	0,11	0,50	0,02	0,14	3	0,19	0,19	0,31	0,49	0,25	1,70	4	1,20	0,95	0,41	0,25	0,07	0,94
6	6	0,10	0,12	0,23	0,53	0,35	2,20	7	0,23	0,39	0,12	0,68	0,11	0,15	6	0,22	0,23	0,30	0,65	0,11	2,10	5	1,90	1,20	0,34	0,61	0,08	0,80
7	5	0,10	0,16	0,38	0,31	0,32	2,50	6	0,30	0,36	0,15	0,62	0,14	1,20	5	0,10	0,23	0,31	0,85	0,35	1,60	4	1,60	1,20	0,30	0,48	0,05	0,60
8	6	0,11	0,12	0,70	0,47	0,33	1,90	7	0,26	0,33	0,09	0,95	0,10	0,01	6	0,24	0,19	0,28	0,43	0,09	1,70	5	0,84	1,30	0,26	0,31	0,07	1,10
9	6	0,09	0,14	0,44	0,62	0,36	2,60	5	0,24	0,36	0,10	0,31	0,15	0,96	6	0,10	0,12	0,36	0,18	0,16	1,80	5	0,97	1,30	0,31	0,63	0,08	0,99
10	5	0,12	0,14	0,36	0,80	0,36	1,60	5	0,24	0,39	0,08	0,56	0,08	0,83	5	0,30	0,27	0,39	0,48	0,25	2,30	5	1,70	1,30	0,37	0,14	0,07	1,10
11	4	0,10	0,13	0,31	0,55	0,33	1,50	6	0,25	0,39	0,14	0,15	0,18	2,00	4	0,21	0,24	0,32	0,28	0,28	0,94	3	1,60	1,00	0,31	0,52	0,07	1,60
12	4	0,09	0,14	0,27	0,46	0,39	2,00	5	0,27	0,40	0,16	0,68	0,10	1,50	4	0,16	0,15	0,35	0,50	0,10	1,80	4	1,70	1,40	0,25	0,28	0,05	0,95
13	6	0,14	0,07	0,58	0,55	0,31	1,50	5	0,22	0,34	0,10	0,37	0,13	0,89	6	0,26	0,21	0,38	0,45	0,28	1,80	4	1,60	1,30	0,31	0,50	0,08	1,60
14	6	0,13	0,10	0,03	0,78	0,38	1,80	8	0,20	0,36	0,13	0,52	0,09	1,30	6	0,12	0,20	0,26	0,20	0,16	1,60	2	1,40	1,40	0,28	0,43	0,09	1,20
15	5	0,13	0,13	0,30	0,42	0,34	1,20	6	0,24	0,37	0,10	0,52	0,14	1,50	5	0,12	0,18	0,33	0,45	0,22	1,70	4	1,70	1,10	0,30	0,80	0,06	0,83
16	6	0,09	0,14	0,09	0,44	0,29	1,60	5	0,20	0,33	0,14	0,54	0,12	1,90	6	0,12	0,26	0,29	0,70	0,25	2,30	5	0,62	0,95	0,32	0,34	0,07	1,40
17	6	0,12	0,13	0,09	0,66	0,26	1,70	6	0,19	0,31	0,13	0,41	0,15	1,10	6	0,22	0,22	0,35	0,39	0,20	2,30	2	0,74	1,50	0,33	0,29	0,08	1,20
18	7	0,14	0,17	0,28	0,83	0,31	1,40	5	0,24	0,38	0,08	0,08	0,12	1,50	7	0,23	0,16	0,32	0,72	0,28	1,80	4	1,20	1,10	0,39	0,51	0,06	1,30
19	4	0,13	0,11	0,35	0,55	0,29	1,80	5	0,27	0,30	0,12	0,17	0,11	0,29	4	0,20	0,24	0,26	0,17	0,23	1,70	3	1,90	0,88	0,34	0,44	0,07	1,00
20	5	0,08	0,14	0,57	0,25	0,28	2,10	7	0,29	0,37	0,12	0,53	0,11	1,90	5	0,21	0,31	0,33	0,46	0,29	1,40	3	0,95	1,70	0,35	0,37	0,06	0,34
21	3	0,12	0,11	0,47	0,47	0,34	2,10	6	0,23	0,38	0,18	0,40	0,09	1,10	3	0,16	0,18	0,28	0,71	0,27	2,10	6	1,50	1,10	0,27	0,11	0,08	0,25
22	7	0,13	0,09	0,03	0,10	0,33	1,60	4	0,24	0,32	0,11	0,31	0,17	1,30	7	0,18	0,20	0,35	0,35	0,18	1,10	4	1,50	1,30	0,34	0,33	0,06	1,20
23	6	0,11	0,13	0,45	0,78	0,30	2,20	6	0,20	0,33	0,10	0,34	0,06	1,20	6	0,20	0,24	0,31	0,59	0,23	2,00	4	1,30	1,00	0,33	0,84	0,09	0,61
24	5	0,11	0,13	0,24	0,90	0,35	2,30	4	0,23	0,40	0,11	0,32	0,11	0,31	5	0,12	0,26	0,33	0,03	0,21	1,80	4	1,50	0,67	0,36	0,77	0,07	1,30
25	5	0,11	0,12	0,24	0,10	0,40	1,10	4	0,23	0,28	0,17	0,27	0,21	0,34	5	0,22	0,22	0,34	0,80	0,21	0,94	5	1,10	1,40	0,31	0,25	0,06	1,30
26	5	0,16	0,08	0,46	0,78	0,32	2,80	7	0,23	0,30	0,19	0,59	0,12	2,40	5	0,17	0,18	0,31	0,41	0,14	2,00	3	1,40	0,92	0,29	0,35	0,07	1,60
27	6	0,08	0,09	0,65	0,40	0,32	0,96	6	0,19	0,29	0,10	0,86	0,19	0,89	6	0,16	0,31	0,34	0,85	0,31	3,10	6	1,10	1,10	0,33	0,36	0,06	1,50
28	5	0,08	0,15	0,21	0,34	0,35	0,64	5	0,26	0,34	0,10	0,23	0,13	1,40	5	0,25	0,17	0,37	0,47	0,06	1,40	3	0,81	1,40	0,37	0,20	0,06	1,30
29	5	0,14	0,11	0,28	0,87	0,34	1,80	6	0,25	0,36	0,09	0,31	0,04	1,20	5	0,16	0,17	0,29	0,49	0,27	1,60	3	0,50	0,87	0,29	0,17	0,05	0,89
30	4	0,13	0,12	0,39	0,32	0,37	0,76	6	0,23	0,32	0,10	0,44	0,12	0,05	4	0,14	0,13	0,30	0,65	0,11	1,80	3	1,60	1,30	0,31	0,21	0,06	0,53

Pat no	time point 36 score	E1	E2	E3	RF	ANA	ACPA	time point 39 score	E1	E2	E3	RF	ANA	ACPA	time point 42 score	E1	E2	E3	RF	ANA	ACPA	time point 45 score	E1	E2	E3	RF	ANA	ACPA
1	8	0,12	0,31	0,04	0,65	0,54	1,60	5,00	0,10	2,20	0,36	0,35	0,06	0,46	2,00	1,80	2,40	0,31	0,15	0,05	2,90	3	0,82	2,80	0,52	0,15	0,09	1,30
2	6	0,09	0,33	0,04	0,53	0,44	0,88	3,90	0,07	2,40	0,37	0,34	0,06	0,92	2,30	2,70	3,30	0,32	0,19	0,08	1,70	3	0,83	1,70	0,04	0,18	0,06	0,75
3	7	0,13	0,36	0,04	0,69	0,45	1,80	3,10	0,09	2,00	0,33	0,17	0,07	0,98	3,60	1,70	3,00	0,34	0,18	0,06	2,00	3	0,37	1,20	0,04	0,22	0,07	0,88
4	6	0,09	0,32	0,04	0,23	0,25	1,60	3,90	0,09	3,10	0,38	0,24	0,07	1,20	1,60	1,90	3,30	0,37	0,11	0,06	0,51	1	0,76	2,20	0,55	0,15	0,09	1,20
5	7	0,06	0,33	0,04	0,46	0,46	0,86	4,20	0,06	2,30	0,41	0,32	0,05	0,84	1,90	1,90	3,10	0,38	0,16	0,06	2,00	2	0,43	1,90	0,04	0,17	0,08	0,54
6	5	0,08	0,32	0,04	0,88	0,39	1,50	4,80	0,07	2,20	0,39	0,27	0,08	1,40	4,40	2,50	3,60	0,34	0,18	0,07	3,00	1	0,93	2,00	0,04	0,15	0,07	1,50
7	5	0,17	0,37	0,04	0,19	0,37	0,98	3,00	0,05	1,90	0,45	0,34	0,06	0,80	3,40	1,60	3,20	0,40	0,15	0,06	1,20	3	0,76	1,20	0,04	0,12	0,07	1,40
8	6	0,08	0,33	0,04	0,52	0,49	1,80	2,90	0,06	1,60	0,35	0,27	0,06	1,90	2,90	2,80	2,50	0,24	0,16	0,07	2,60	2	0,85	1,90	0,04	0,11	0,07	1,10
9	7	0,08	0,34	0,04	0,89	0,29	1,60	2,60	0,07	2,10	0,34	0,29	0,09	1,10	2,60	1,70	3,20	0,40	0,16	0,08	1,80	4	0,41	1,60	0,04	0,15	0,10	0,87
10	7	0,13	0,35	0,04	0,56	0,71	1,60	3,30	0,08	2,20	0,32	0,24	0,08	0,70	2,20	2,50	2,60	0,32	0,20	0,08	1,50	1	1,10	1,00	0,04	0,22	0,08	0,90
11	5	0,09	0,33	0,04	0,54	0,48	1,90	4,20	0,07	1,90	0,34	0,24	0,08	0,98	3,20	2,00	3,20	0,36	0,13	0,08	2,60	1	0,75	1,60	0,04	0,16	0,05	0,56
12	6	0,12	0,31	0,04	0,51	0,39	0,78	5,30	0,05	2,10	0,39	0,39	0,07	1,50	4,60	2,20	2,80	0,35	0,11	0,07	2,40	3	1,00	2,40	0,04	0,20	0,07	1,10
13	7	0,15	0,34	0,04	0,60	0,31	0,87	4,50	0,06	2,80	0,34	0,27	0,06	1,30	2,70	3,00	2,40	0,42	0,12	0,07	2,00	3	0,74	1,60	0,04	0,15	0,07	1,40
14	5	0,15	0,33	0,04	0,61	0,65	0,58	4,40	0,07	2,20	0,40	0,18	0,08	1,10	4,10	2,50	2,50	0,28	0,13	0,05	0,97	2	0,68	1,50	0,04	0,16	0,09	1,70
15	6	0,12	0,36	0,04	0,57	0,44	1,70	4,90	0,08	2,10	0,39	0,18	0,07	0,80	2,30	2,30	2,20	0,35	0,13	0,05	1,80	2	0,57	1,10	0,04	0,16	0,06	1,20
16	6	0,08	0,36	0,04	0,34	0,51	1,10	2,20	0,07	2,40	0,37	0,31	0,08	1,30	2,10	1,70	2,90	0,34	0,12	0,08	1,40	0	0,81	2,50	0,04	0,15	0,07	1,40
17	8	0,12	0,36	0,04	0,25	0,52	0,72	3,40	0,07	2,10	0,31	0,12	0,06	1,30	2,60	1,40	3,30	0,40	0,13	0,08	1,10	2	0,76	1,50	0,04	0,14	0,08	0,44
18	7	0,14	0,34	0,04	0,48	0,32	0,99	3,80	0,05	1,90	0,38	0,28	0,08	0,75	3,20	3,00	2,50	0,35	0,14	0,09	1,30	2	0,85	1,20	0,04	0,17	0,08	0,45
19	8	0,07	0,35	0,04	0,36	0,40	0,48	4,70	0,08	2,30	0,36	0,29	0,06	0,74	2,10	2,80	2,90	0,26	0,18	0,07	0,92	3	0,60	2,30	0,04	0,14	0,05	0,44
20	6	0,11	0,35	0,04	0,54	0,54	0,63	3,20	0,08	2,40	0,30	0,39	0,07	0,75	3,40	3,00	2,90	0,32	0,12	0,06	2,80	1	0,55	1,80	0,04	0,12	0,06	0,60
21	8	0,15	0,34	0,04	0,36	0,39	0,69	2,50	0,05	1,80	0,27	0,25	0,07	0,94	3,90	2,80	3,50	0,36	0,15	0,08	1,10	1	1,10	2,10	0,04	0,17	0,07	0,89
22	5	0,05	0,32	0,04	0,69	0,36	0,17	5,20	0,08	2,70	0,37	0,33	0,07	0,57	4,10	1,70	2,70	0,37	0,19	0,08	2,20	2	1,10	1,10	0,04	0,16	0,06	1,10
23	6	0,10	0,31	0,04	0,79	0,32	1,40	4,20	0,09	1,50	0,35	0,22	0,06	0,89	2,20	1,40	2,40	0,36	0,15	0,08	0,73	1	0,49	1,70	0,04	0,13	0,07	1,30
24	7	0,11	0,37	0,04	0,61	0,49	1,20	3,70	0,05	2,50	0,27	0,23	0,06	0,80	4,00	1,80	2,30	0,35	0,14	0,07	0,99	3	0,55	1,80	0,04	0,13	0,08	1,10
25	4	0,09	0,35	0,04	0,58	0,35	0,59	3,60	0,06	1,70	0,39	0,28	0,09	1,60	3,90	1,60	1,80	0,36	0,19	0,07	1,80	3	0,92	2,20	0,04	0,16	0,08	0,18
26	7	0,19	0,33	0,04	0,26	0,38	1,10	4,30	0,07	1,60	0,27	0,33	0,06	0,97	1,70	2,00	2,40	0,30	0,18	0,07	1,70	1	0,91	1,30	0,04	0,15	0,07	1,10
27	7	0,16	0,35	0,04	0,31	0,64	1,20	3,50	0,03	1,80	0,38	0,25	0,07	1,20	2,80	2,00	3,00	0,35	0,12	0,08	2,70	2	0,62	1,90	0,04	0,14	0,09	0,69
28	8	0,09	0,33	0,04	0,57	0,44	1,70	2,90	0,06	2,40	0,41	0,30	0,07	1,40	2,60	2,60	3,00	0,37	0,18	0,06	2,60	1	0,65	0,73	0,04	0,13	0,08	1,20
29	5	0,12	0,35	0,04	0,59	0,49	0,83	3,40	0,04	1,90	0,28	0,20	0,09	1,60	1,30	2,20	1,60	0,38	0,16	0,10	2,60	2	0,49	2,00	0,04	0,15	0,07	0,93
30	7	0,12	0,35	0,04	0,40	0,59	1,00	4,10	0,05	1,40	0,33	0,30	0,05	1,50	3,10	2,10	2,40	0,36	0,10	0,07	1,60	2	0,94	0,89	0,04	0,15	0,08	0,98

Pat no	time point 48 score	E1	E2	E3	RF	ANA	ACPA	time point 51 score	E1	E2	E3	RF	ANA	ACPA
1	7	1,20	1,30	0,11	0,10	0,09	1,50	4,50	0,08	1,40	0,32	0,02	0,07	1,10
2	7	1,30	1,40	0,07	0,13	0,07	1,50	4,80	0,11	1,60	0,14	0,16	0,10	1,50
3	7	0,80	1,40	0,06	0,06	0,08	0,75	3,80	0,12	1,30	0,24	0,10	0,10	1,20
4	6	1,30	1,20	0,08	0,10	0,09	1,80	3,00	0,10	1,50	0,15	0,12	0,05	0,88
5	6	1,30	1,40	0,07	0,15	0,09	0,71	4,40	0,11	1,30	0,22	0,04	0,08	1,30
6	6	1,20	1,50	0,07	0,09	0,07	1,00	3,30	0,08	1,40	0,25	0,00	0,07	1,60
7	6	0,78	1,20	0,07	0,15	0,08	0,87	3,90	0,09	1,40	0,25	0,14	0,07	0,88
8	7	0,93	1,40	0,12	0,10	0,09	1,70	1,90	0,11	1,40	0,37	0,02	0,08	0,51
9	6	1,80	1,30	0,10	0,13	0,06	1,70	3,90	0,09	1,50	0,23	0,14	0,09	0,64
10	6	1,40	1,10	0,15	0,01	0,08	1,00	4,30	0,13	1,50	0,25	0,10	0,09	1,50
11	5	0,53	1,40	0,15	0,12	0,07	1,60	2,90	0,13	1,30	0,06	0,12	0,07	0,90
12	4	0,88	1,40	0,11	0,10	0,08	0,47	4,30	0,09	1,30	0,22	0,10	0,10	1,40
13	6	1,20	1,20	0,13	0,09	0,07	0,75	3,60	0,12	1,40	0,22	0,10	0,09	1,10
14	5	1,60	1,40	0,16	0,06	0,09	1,00	2,90	0,09	1,40	0,24	0,10	0,07	1,60
15	7	0,58	1,20	0,12	0,15	0,10	1,20	4,00	0,12	1,50	0,17	0,05	0,07	0,86
16	6	1,20	1,30	0,13	0,13	0,09	1,90	4,10	0,12	1,40	0,18	0,06	0,07	1,20
17	7	1,10	1,20	0,11	0,14	0,07	1,10	3,40	0,08	1,50	0,33	0,13	0,09	1,00
18	7	0,89	1,40	0,14	0,09	0,08	1,20	1,50	0,13	1,50	0,24	0,12	0,10	1,30
19	6	1,50	1,40	0,14	0,08	0,07	1,30	4,70	0,08	1,60	0,21	0,05	0,09	1,40
20	8	1,40	1,30	0,08	0,19	0,10	1,40	4,70	0,12	1,40	0,26	0,11	0,08	0,97
21	5	0,96	1,30	0,07	0,11	0,08	0,69	2,60	0,08	1,60	0,32	0,06	0,09	1,60
22	6	0,61	1,40	0,18	0,08	0,07	1,30	5,30	0,07	1,50	0,25	0,07	0,09	1,30
23	7	1,30	1,30	0,05	0,11	0,10	2,00	3,90	0,08	1,50	0,30	0,05	0,09	1,30
24	5	1,50	1,30	0,09	0,14	0,09	0,73	6,50	0,08	1,30	0,34	0,07	0,07	1,10
25	5	1,30	1,30	0,10	0,14	0,09	0,55	3,90	0,11	1,30	0,25	0,11	0,10	0,92
26	6	1,70	1,30	0,14	0,13	0,10	0,90	4,00	0,12	1,40	0,27	0,14	0,09	0,96
27	7	1,60	1,30	0,11	0,07	0,07	1,10	3,70	0,12	1,40	0,40	0,05	0,10	1,20
28	4	1,60	1,20	0,07	0,16	0,07	1,60	3,90	0,09	1,60	0,27	0,07	0,05	1,20
29	4	1,60	1,30	0,13	0,13	0,08	1,00	3,90	0,08	1,50	0,26	0,07	0,09	0,97
30	7	1,60	1,30	0,10	0,08	0,08	0,09	3,50	0,09	1,60	0,21	0,14	0,08	1,70

Pat no	time point 54 score	E1	E2	E3	RF	ANA	ACPA	time point 57 score	E1	E2	E3	RF	ANA	ACPA
1	5,10	0,18	2,60	0,11	0,03	0,09	1,50	5	0,11	2,00	0,15	0,30	0,23	1,50
2	5,70	0,24	2,80	0,26	0,05	0,08	0,92	7	0,11	1,30	0,08	0,19	0,24	0,98
3	6,20	0,23	2,60	0,22	0,11	0,08	1,10	5	0,11	2,20	0,17	0,24	0,25	0,23
4	4,50	0,22	2,00	0,11	0,03	0,07	1,50	6	0,12	1,80	0,10	0,26	0,23	1,20
5	7,50	0,20	2,40	0,29	0,03	0,07	1,00	6	0,13	2,60	0,14	0,35	0,23	0,67
6	5,00	0,19	1,30	0,17	0,04	0,09	1,70	8	0,12	1,10	0,15	0,28	0,24	0,56
7	7,50	0,21	2,50	0,19	0,08	0,06	1,30	5	0,09	2,20	0,15	0,24	0,24	1,30
8	5,60	0,22	1,90	0,26	0,05	0,07	1,00	5	0,12	1,50	0,11	0,29	0,24	1,10
9	7,00	0,21	2,70	0,38	0,08	0,07	1,50	6	0,14	0,50	0,16	0,20	0,23	1,10
10	4,20	0,22	2,00	0,15	0,15	0,06	1,00	6	0,10	0,96	0,09	0,30	0,25	0,80
11	7,00	0,23	2,50	0,20	0,08	0,08	1,20	8	0,12	1,40	0,20	0,21	0,24	0,93
12	6,70	0,22	2,60	0,25	0,10	0,09	1,30	5	0,12	0,90	0,12	0,21	0,24	1,10
13	7,70	0,20	2,50	0,26	0,11	0,07	1,30	6	0,13	2,30	0,09	0,25	0,25	1,70
14	7,20	0,21	3,40	0,13	0,01	0,07	0,78	6	0,14	2,30	0,03	0,18	0,25	1,30
15	4,30	0,21	2,70	0,27	0,10	0,09	1,50	7	0,11	1,70	0,16	0,28	0,24	1,10
16	5,40	0,19	1,90	0,31	0,09	0,09	0,67	6	0,13	1,10	0,15	0,33	0,25	0,75
17	5,10	0,25	2,70	0,22	0,04	0,08	0,80	6	0,11	1,70	0,14	0,18	0,25	0,53
18	4,30	0,19	1,70	0,35	0,07	0,08	1,40	6	0,14	1,90	0,07	0,25	0,25	1,10
19	6,70	0,21	3,10	0,26	0,12	0,09	1,60	6	0,11	1,20	0,09	0,26	0,25	1,60
20	5,00	0,21	3,20	0,17	0,05	0,08	0,98	6	0,14	1,30	0,11	0,28	0,24	0,85
21	6,00	0,15	2,10	0,12	0,09	0,07	1,20	6	0,14	1,50	0,18	0,32	0,25	0,42
22	5,30	0,22	2,10	0,25	0,12	0,09	1,40	7	0,13	2,70	0,16	0,13	0,22	0,95
23	8,00	0,24	2,50	0,12	0,10	0,07	0,77	8	0,11	2,10	0,12	0,17	0,24	1,40
24	5,50	0,23	3,00	0,25	0,08	0,08	0,55	7	0,12	1,20	0,15	0,26	0,24	1,30
25	5,80	0,21	2,90	0,24	0,14	0,09	1,30	8	0,10	1,50	0,09	0,28	0,24	1,10
26	7,80	0,21	2,50	0,26	0,07	0,10	1,40	6	0,11	2,30	0,12	0,14	0,22	1,10
27	6,30	0,24	2,80	0,07	0,08	0,09	1,10	6	0,12	1,50	0,13	0,20	0,24	1,30
28	5,60	0,18	4,20	0,17	0,14	0,09	1,60	7	0,11	1,10	0,17	0,25	0,24	0,36
29	7,10	0,23	3,60	0,14	0,09	0,08	1,60	6	0,12	2,20	0,16	0,27	0,24	1,60
30	6,30	0,19	2,40	0,36	0,10	0,09	1,00	5	0,14	2,50	0,17	0,14	0,24	0,71

Pat no	time point 60 score	E1	E2	E3	RF	ANA	ACPA
1	4	0,27	2,30	0,72	0,21	0,23	0,61
2	6	0,25	2,10	0,61	0,21	0,20	1,20
3	5	0,21	1,70	0,66	0,22	0,22	2,20
4	6	0,24	2,40	0,77	0,08	0,21	2,20
5	5	0,23	2,00	0,65	0,18	0,21	2,00
6	4	0,24	2,70	0,79	0,16	0,22	2,10
7	5	0,23	1,80	0,69	0,22	0,21	2,10
8	2	0,21	2,60	0,62	0,14	0,22	1,80
9	4	0,22	1,60	0,64	0,12	0,20	1,50
10	3	0,21	2,30	0,43	0,24	0,22	1,40
11	8	0,24	2,30	0,72	0,14	0,20	2,60
12	5	0,21	1,30	0,65	0,18	0,21	3,10
13	6	0,26	2,40	0,74	0,19	0,18	2,11
14	6	0,23	1,80	0,72	0,22	0,20	0,61
15	7	0,23	2,00	0,68	0,35	0,21	1,20
16	6	0,22	3,10	0,86	0,21	0,21	3,00
17	4	0,25	2,00	0,70	0,27	0,21	1,70
18	6	0,23	2,50	0,76	0,24	0,19	2,20
19	6	0,21	2,40	0,85	0,23	0,21	1,50
20	6	0,20	1,90	0,65	0,16	0,20	2,00
21	4	0,23	2,00	0,59	0,21	0,21	1,60
22	7	0,22	2,30	0,79	0,24	0,22	1,11
23	8	0,23	2,60	0,46	0,15	0,20	0,35
24	7	0,23	1,70	0,70	0,12	0,21	1,60
25	8	0,22	1,80	0,70	0,26	0,21	1,70
26	6	0,21	2,22	0,56	0,22	0,21	2,20
27	6	0,24	1,60	0,24	0,24	0,18	1,50
28	7	0,21	2,00	0,65	0,25	0,21	1,45
29	4	0,26	2,30	0,59	0,12	0,20	1,60
30	2	0,23	1,30	0,44	0,11	0,19	2,00

ELISA has been carried out as described in Methods. Randomly selected 5% of samples have been tested in triplicate using the same plate. According to chi2 statistical test, 5% have been a sufficient sampling to represent the entire cohort (30 out of 600 total observations for each test; chi-square statistic is 33.2908. The p-value is < 0.00001). CV values were within the range given in Methods section. Below, individual data points for all subjects are given.
