# Supplementary material for: Antibodies to synthetic citrullinated peptide epitope correlate with disease activity and flares in rheumatoid arthritis
Source: PLoS One. 2020 Apr 23;15(4):e0232010. doi: 10.1371/journal.pone.0232010 (PMC7179858; doi:10.1371/journal.pone.0232010)
Supplement: S7 Appendix — S10 Table. Mean A450 values, SLE cohort (N = 30). S11. Individual data points for longitudinal study, SLE cohort. (PDF) [file pone.0232010.s007.pdf]

S7 Appendix. Mean A450 and individual A450 values, SLE cohort.

S10 Table. Mean A450 values, SLE cohort (N = 30)

time, months	mean SLEDAI	E1	E2	E3	RF	ANA	ACPA
0	6	0.06	0.07	0.47	0.06	1.7	0.51
3	5	0.11	0.41	0.45	0.11	1.44	0.49
6	5	0.34	0.13	0.02	0.36	1.13	0.1
9	3	0.14	0.46	0.12	0.15	0.5	0.12
12	4	0.48	0.29	0.32	2.77	0.31	0.35
15	2	0.28	0.12	0.11	1.2	0.12	0.11
18	4	0.29	0.06	0.04	0.11	0.06	0.32
21	5	0.48	0.11	3.08	0.28	0.11	0.44
24	4	0.15	0.34	0.11	0.43	0.37	0.11
27	6	0.32	0.15	0.13	0.21	1.55	0.13
30	7	0.04	0.1	0.1	0.04	2.1	0.1
33	4	0.43	1.11	0.07	0.47	1.23	0.06
36	5	0.65	0.28	0.28	0.72	0.3	0.3
39	3	0.05	0.29	0.09	0.04	0.31	0.09
42	2	0.03	0.49	2.87	0.02	0.53	0.22
45	2	0.05	0.28	0.13	0.04	0.3	0.13
48	3	0.11	0.09	0.3	0.11	1.9	0.33
51	4	0.07	1.18	0.24	0.06	1.31	0.25
54	6	0.09	0.13	0.32	0.08	2.13	0.35
57	6	0.31	0.3	0.11	0.34	2.1	0.11
60	4	0.25	0.24	0.04	0.26	1.25	0.3

S11. Individual data points for longitudinal study, SLE cohort.

Pat no	time point 0							time point 3							time point 6							time point 9						
	score	E1	E2	E3	RF	ANA	ACPA	score	E1	E2	E3	RF	ANA	ACPA	score	E1	E2	E3	RF	ANA	ACPA	score	E1	E2	E3	RF	ANA	ACPA
1	5	0,06	0,07	0,46	0,07	2,20	0,47	5	0,10	0,27	0,52	0,05	1,60	0,25	5	0,35	0,14	0,02	0,28	1,30	0,14	5	0,14	0,42	0,13	0,08	0,51	0,10
2	6	0,07	0,06	0,46	0,07	1,30	0,39	5	0,11	0,43	0,48	0,08	1,10	0,40	5	0,34	0,10	0,03	0,27	0,47	0,18	3	0,15	0,42	0,04	0,17	0,49	0,08
3	5	0,06	0,06	0,52	0,06	1,90	0,63	5	0,13	0,40	0,47	0,18	1,30	0,41	4	0,35	0,09	0,02	0,41	0,72	0,06	3	0,13	0,43	0,13	0,17	0,51	0,08
4	7	0,05	0,06	0,43	0,06	1,60	0,49	5	0,11	0,32	0,44	0,24	1,30	0,46	4	0,35	0,15	0,02	0,42	0,45	0,19	3	0,13	0,46	0,08	0,20	0,48	0,17
5	7	0,04	0,05	0,45	0,06	2,40	0,39	5	0,10	0,38	0,45	0,11	2,00	0,27	5	0,35	0,11	0,03	0,44	1,20	0,10	2	0,12	0,44	0,11	0,20	0,50	0,02
6	6	0,07	0,06	0,50	0,06	1,50	0,43	4	0,09	0,42	0,46	0,10	1,80	0,40	4	0,34	0,12	0,02	0,30	1,40	0,09	3	0,15	0,46	0,09	0,19	0,49	0,12
7	7	0,06	0,07	0,44	0,07	1,70	0,44	5	0,12	0,33	0,49	0,02	1,60	0,29	5	0,34	0,14	0,02	0,36	1,20	0,05	2	0,13	0,46	0,09	0,19	0,48	0,17
8	5	0,05	0,08	0,47	0,05	1,40	0,42	6	0,11	0,43	0,41	0,15	1,50	0,38	5	0,34	0,15	0,02	0,39	1,30	0,08	5	0,15	0,44	0,17	0,14	0,51	0,09
9	6	0,07	0,07	0,51	0,06	1,20	0,51	7	0,12	0,37	0,48	0,02	1,60	0,44	5	0,36	0,16	0,02	0,33	1,60	0,04	3	0,15	0,46	0,13	0,02	0,49	0,04
10	4	0,06	0,08	0,46	0,06	1,90	0,39	5	0,11	0,38	0,51	0,14	0,77	0,48	7	0,33	0,10	0,03	0,32	0,95	0,19	2	0,14	0,45	0,02	0,16	0,52	0,03
11	7	0,05	0,04	0,57	0,06	1,80	0,49	6	0,10	0,35	0,50	0,02	0,92	0,38	4	0,35	0,14	0,02	0,33	1,20	0,16	5	0,14	0,45	0,10	0,10	0,52	0,09
12	5	0,07	0,09	0,44	0,07	1,50	0,59	4	0,10	0,50	0,49	0,06	1,50	0,45	6	0,32	0,13	0,02	0,17	1,70	0,08	4	0,15	0,39	0,19	0,22	0,48	0,21
13	6	0,08	0,06	0,50	0,06	2,20	0,48	8	0,10	0,48	0,49	0,16	1,30	0,43	5	0,33	0,16	0,02	0,32	0,64	0,15	3	0,14	0,44	0,18	0,11	0,51	0,23
14	6	0,06	0,08	0,43	0,05	0,98	0,62	5	0,10	0,41	0,47	0,19	1,70	0,43	4	0,33	0,11	0,02	0,52	1,10	0,06	3	0,15	0,46	0,18	0,13	0,52	0,02
15	5	0,06	0,08	0,43	0,06	1,50	0,45	4	0,10	0,41	0,50	0,08	1,10	0,38	2	0,35	0,10	0,02	0,40	0,95	0,11	3	0,15	0,48	0,10	0,14	0,49	0,13
16	6	0,08	0,06	0,45	0,07	1,00	0,50	3	0,11	0,38	0,55	0,15	1,60	0,37	5	0,35	0,15	0,03	0,36	1,10	0,14	2	0,15	0,46	0,07	0,10	0,49	0,05
17	5	0,06	0,08	0,42	0,07	2,00	0,45	5	0,10	0,31	0,49	0,03	1,20	0,45	5	0,35	0,12	0,02	0,43	0,88	0,05	4	0,12	0,47	0,01	0,10	0,49	0,21
18	6	0,06	0,07	0,42	0,06	2,50	0,68	3	0,12	0,44	0,54	0,04	1,30	0,45	3	0,36	0,07	0,02	0,41	0,71	0,08	3	0,14	0,45	0,10	0,22	0,50	0,19
19	6	0,07	0,08	0,50	0,07	1,20	0,57	5	0,12	0,39	0,52	0,13	2,10	0,36	5	0,33	0,11	0,02	0,36	1,50	0,01	3	0,14	0,47	0,11	0,10	0,49	0,07
20	6	0,06	0,08	0,42	0,07	1,40	0,56	4	0,12	0,44	0,42	0,19	1,50	0,24	6	0,35	0,14	0,02	0,33	1,30	0,06	3	0,15	0,45	0,08	0,15	0,50	0,14
21	7	0,06	0,07	0,46	0,06	2,80	0,51	7	0,11	0,48	0,48	0,04	1,90	0,18	7	0,33	0,08	0,02	0,37	1,00	0,10	4	0,16	0,42	0,17	0,24	0,52	0,13
22	6	0,06	0,08	0,49	0,06	1,80	0,66	4	0,09	0,38	0,48	0,12	1,30	0,38	4	0,36	0,09	0,02	0,38	0,75	0,04	3	0,13	0,42	0,19	0,21	0,54	0,11
23	6	0,04	0,06	0,50	0,07	2,20	0,60	4	0,11	0,40	0,43	0,18	1,20	0,53	4	0,33	0,15	0,02	0,46	1,30	0,06	3	0,15	0,48	0,07	0,10	0,51	0,02
24	6	0,06	0,07	0,47	0,06	1,40	0,64	4	0,11	0,41	0,46	0,05	1,70	0,37	6	0,35	0,15	0,01	0,35	1,50	0,21	5	0,13	0,43	0,10	0,13	0,51	0,18
25	6	0,06	0,06	0,46	0,06	1,30	0,52	7	0,11	0,35	0,48	0,00	1,30	0,48	4	0,34	0,15	0,02	0,40	1,10	0,09	4	0,13	0,44	0,20	0,14	0,52	0,01
26	6	0,05	0,09	0,46	0,06	0,56	0,58	7	0,11	0,35	0,41	0,11	1,30	0,43	4	0,34	0,07	0,02	0,28	1,20	0,07	5	0,15	0,52	0,06	0,16	0,52	0,09
27	7	0,08	0,07	0,44	0,07	0,99	0,35	5	0,10	0,31	0,50	0,20	1,10	0,40	5	0,34	0,17	0,02	0,42	0,65	0,09	3	0,13	0,47	0,09	0,15	0,47	0,14
28	6	0,05	0,08	0,43	0,06	2,10	0,71	3	0,11	0,39	0,55	0,12	1,10	0,55	4	0,34	0,15	0,02	0,32	1,10	0,12	4	0,14	0,43	0,19	0,24	0,49	0,06
29	8	0,05	0,07	0,46	0,05	2,10	0,57	5	0,10	0,45	0,45	0,19	1,80	0,32	5	0,34	0,16	0,02	0,24	1,40	0,11	3	0,14	0,42	0,14	0,15	0,50	0,16
30	6	0,07	0,06	0,42	0,06	1,20	0,38	7	0,11	0,46	0,50	0,19	1,50	0,59	3	0,32	0,15	0,03	0,29	0,77	0,10	2	0,14	0,49	0,07	0,21	0,54	0,15

Pat no	time point 12							time point 15							time point 18							time point 21						
	score	E1	E2	E3	RF	ANA	ACPA	score	E1	E2	E3	RF	ANA	ACPA	score	E1	E2	E3	RF	ANA	ACPA	score	E1	E2	E3	RF	ANA	ACPA
1	4	0,53	0,27	0,30	3,80	0,36	0,35	2	0,36	0,12	0,13	1,10	0,13	0,14	5	0,13	0,06	0,03	0,12	0,11	0,21	3	0,50	0,11	3,00	0,24	0,14	0,51
2	2	0,46	0,30	0,22	2,20	0,22	0,36	3	0,29	0,01	0,21	0,28	0,11	0,22	5	0,20	0,05	0,04	0,10	0,05	0,22	4	0,50	0,11	3,80	0,33	0,12	0,49
3	3	0,58	0,27	0,31	3,20	0,14	0,39	2	0,22	0,11	0,13	1,60	0,12	0,10	2	0,20	0,07	0,05	0,16	0,02	0,27	3	0,49	0,11	2,80	0,28	0,08	0,31
4	4	0,41	0,34	0,39	2,70	0,29	0,33	2	0,34	0,15	0,12	1,60	0,09	0,03	3	0,26	0,07	0,04	0,05	0,08	0,30	4	0,45	0,11	3,70	0,23	0,13	0,40
5	3	0,48	0,25	0,37	2,90	0,27	0,42	3	0,26	0,10	0,10	1,20	0,12	0,14	4	0,23	0,06	0,01	0,10	0,05	0,23	3	0,47	0,11	3,00	0,30	0,10	0,48
6	5	0,38	0,33	0,21	3,10	0,38	0,37	2	0,27	0,12	0,17	1,10	0,11	0,09	4	0,31	0,07	0,05	0,08	0,04	0,25	3	0,38	0,10	2,80	0,29	0,11	0,51
7	4	0,46	0,25	0,39	2,10	0,35	0,37	2	0,34	0,10	0,10	0,15	0,13	0,06	5	0,31	0,05	0,06	0,05	0,08	0,25	5	0,49	0,11	3,60	0,19	0,12	0,64
8	5	0,47	0,27	0,24	1,20	0,30	0,38	2	0,37	0,16	0,07	1,40	0,12	0,20	6	0,30	0,05	0,05	0,09	0,03	0,34	6	0,48	0,11	2,80	0,28	0,12	0,49
9	3	0,33	0,26	0,34	2,10	0,31	0,51	3	0,17	0,13	0,18	0,71	0,10	0,16	5	0,34	0,06	0,04	0,08	0,09	0,39	4	0,50	0,11	3,90	0,32	0,10	0,34
10	5	0,44	0,27	0,35	3,70	0,19	0,39	1	0,23	0,02	0,15	0,39	0,15	0,05	5	0,35	0,06	0,05	0,07	0,06	0,34	4	0,39	0,11	3,40	0,18	0,13	0,45
11	5	0,40	0,29	0,22	3,80	0,34	0,24	1	0,22	0,08	0,18	1,30	0,11	0,09	4	0,36	0,07	0,04	0,10	0,04	0,33	5	0,48	0,10	3,00	0,34	0,13	0,46
12	4	0,57	0,37	0,34	2,60	0,40	0,40	1	0,25	0,07	0,14	1,40	0,12	0,04	3	0,24	0,06	0,03	0,11	0,05	0,22	3	0,46	0,11	3,00	0,30	0,15	0,61
13	2	0,51	0,25	0,32	3,60	0,32	0,32	2	0,28	0,10	0,12	1,00	0,10	0,13	3	0,23	0,06	0,06	0,07	0,07	0,43	3	0,63	0,11	2,70	0,23	0,15	0,21
14	5	0,51	0,27	0,36	2,20	0,31	0,46	2	0,26	0,20	0,13	0,96	0,14	0,03	3	0,38	0,06	0,05	0,08	0,09	0,28	3	0,54	0,11	3,10	0,36	0,11	0,28

15	3	0,53	0,19	0,28	1,50	0,34	0,27	2	0,31	0,05	0,08	1,30	0,10	0,12	5	0,29	0,06	0,03	0,12	0,04	0,39	5	0,53	0,11	3,90	0,25	0,13	0,24
16	5	0,46	0,29	0,28	2,20	0,38	0,37	2	0,34	0,13	0,10	1,20	0,11	0,11	3	0,32	0,07	0,05	0,13	0,07	0,32	5	0,48	0,11	2,10	0,32	0,12	0,70
17	5	0,46	0,29	0,37	3,00	0,36	0,33	3	0,28	0,20	0,08	1,30	0,16	0,12	5	0,31	0,06	0,04	0,09	0,06	0,39	6	0,40	0,11	2,60	0,29	0,10	0,44
18	2	0,49	0,34	0,42	2,40	0,32	0,41	1	0,42	0,08	0,15	0,76	0,12	0,11	5	0,33	0,06	0,04	0,05	0,04	0,39	4	0,63	0,11	2,90	0,32	0,13	0,61
19	4	0,50	0,42	0,30	2,80	0,36	0,30	1	0,29	0,05	0,08	1,10	0,14	0,08	5	0,29	0,06	0,03	0,14	0,03	0,34	4	0,45	0,11	4,10	0,33	0,12	0,21
20	2	0,45	0,21	0,33	2,90	0,24	0,48	3	0,27	0,12	0,10	0,87	0,16	0,13	3	0,20	0,06	0,03	0,10	0,07	0,40	5	0,53	0,10	2,80	0,24	0,10	0,51
21	5	0,46	0,22	0,26	2,60	0,29	0,25	3	0,18	0,21	0,19	2,00	0,11	0,04	4	0,24	0,06	0,02	0,11	0,06	0,37	6	0,42	0,11	3,60	0,32	0,10	0,13
22	5	0,53	0,34	0,28	2,60	0,30	0,31	2	0,23	0,14	0,10	2,00	0,10	0,12	5	0,17	0,06	0,04	0,13	0,07	0,21	3	0,47	0,11	3,30	0,28	0,15	0,64
23	4	0,43	0,29	0,24	2,90	0,29	0,32	1	0,29	0,04	0,09	0,66	0,13	0,17	4	0,24	0,06	0,04	0,11	0,06	0,37	4	0,43	0,11	2,80	0,20	0,09	0,49
24	3	0,49	0,35	0,28	3,10	0,29	0,29	0	0,32	0,10	0,20	1,30	0,09	0,15	5	0,27	0,06	0,01	0,05	0,07	0,28	3	0,43	0,11	3,00	0,30	0,14	0,51
25	4	0,51	0,28	0,33	2,90	0,33	0,35	3	0,26	0,19	0,14	1,40	0,11	0,07	3	0,27	0,07	0,06	0,10	0,08	0,33	4	0,52	0,11	2,10	0,26	0,11	0,25
26	4	0,45	0,31	0,36	2,40	0,19	0,33	2	0,26	0,03	0,11	1,10	0,10	0,17	6	0,38	0,06	0,05	0,13	0,08	0,24	3	0,54	0,11	2,80	0,37	0,14	0,40
27	3	0,52	0,44	0,28	3,40	0,31	0,39	1	0,26	0,08	0,19	1,20	0,08	0,20	5	0,33	0,07	0,02	0,10	0,07	0,25	5	0,56	0,11	3,10	0,32	0,15	0,64
28	4	0,60	0,25	0,32	3,20	0,27	0,53	1	0,23	0,10	0,14	1,40	0,14	0,21	5	0,46	0,06	0,06	0,10	0,08	0,31	6	0,44	0,12	3,40	0,18	0,12	0,43
29	3	0,42	0,21	0,28	3,10	0,29	0,24	2	0,27	0,07	0,21	1,80	0,14	0,14	4	0,20	0,07	0,03	0,16	0,11	0,26	5	0,61	0,11	2,70	0,34	0,12	0,13
30	4	0,50	0,32	0,28	3,20	0,33	0,27	0	0,31	0,12	0,08	1,30	0,13	0,06	4	0,26	0,07	0,06	0,03	0,04	0,31	4	0,55	0,11	3,00	0,38	0,09	0,49

Pat no	time point 24							time point 27							time point 30							time point 33						
	score	E1	E2	E3	RF	ANA	ACPA	score	E1	E2	E3	RF	ANA	ACPA	score	E1	E2	E3	RF	ANA	ACPA	score	E1	E2	E3	RF	ANA	ACPA
1	3	0,14	0,34	0,19	0,34	0,33	0,11	6	0,28	0,16	0,01	0,22	2,20	0,11	6	0,04	0,05	0,12	0,04	2,30	0,16	5	0,04	1,50	0,10	0,30	1,00	0,15
2	6	0,17	0,29	0,11	0,58	0,30	0,14	5	0,33	0,25	0,16	0,21	2,30	0,05	7	0,04	0,09	0,20	0,04	2,00	0,09	2	0,04	1,50	0,05	0,57	1,50	0,09
3	3	0,24	0,33	0,09	0,54	0,25	0,10	6	0,32	0,15	0,13	0,20	1,50	0,09	7	0,04	0,30	0,07	0,04	1,70	0,16	3	0,04	0,57	0,02	0,43	1,70	0,09
4	5	0,11	0,36	0,22	0,30	0,48	0,10	5	0,31	0,01	0,13	0,21	1,40	0,12	6	0,04	0,13	0,05	0,04	3,10	0,13	3	0,04	1,60	0,10	0,38	1,50	0,02
5	4	0,21	0,22	0,13	0,60	0,36	0,08	6	0,34	0,12	0,29	0,20	1,70	0,01	6	0,04	0,18	0,13	0,04	2,00	0,18	4	0,04	0,79	0,12	0,46	1,50	0,08
6	5	0,19	0,27	0,03	0,42	0,37	0,06	7	0,34	0,19	0,15	0,21	1,70	0,13	5	0,05	0,18	0,20	0,04	2,60	0,14	3	0,03	1,30	0,17	0,58	2,00	0,13
7	3	0,20	0,29	0,16	0,53	0,36	0,10	5	0,28	0,18	0,03	0,20	1,30	0,05	5	0,04	0,11	0,14	0,04	1,80	0,12	4	0,04	0,45	0,06	0,31	1,50	0,08
8	4	0,14	0,41	0,04	0,47	0,37	0,19	6	0,39	0,23	0,10	0,23	0,61	0,01	7	0,04	0,14	0,11	0,04	2,10	0,10	6	0,04	1,90	0,12	0,48	2,10	0,17
9	2	0,12	0,26	0,11	0,41	0,25	0,18	6	0,33	0,14	0,12	0,22	1,30	0,12	7	0,04	0,20	0,11	0,04	2,60	0,08	4	0,04	1,60	0,09	0,26	1,40	0,11
10	4	0,09	0,35	0,05	0,39	0,35	0,15	4	0,40	0,23	0,14	0,20	1,80	0,11	7	0,04	0,13	0,15	0,04	1,60	0,06	5	0,04	0,16	0,11	0,46	1,70	0,12
11	2	0,17	0,33	0,09	0,55	0,39	0,11	7	0,27	0,18	0,13	0,23	1,70	0,18	5	0,04	0,18	0,06	0,04	2,10	0,08	5	0,04	1,30	0,15	0,37	1,40	0,15
12	4	0,13	0,32	0,14	0,46	0,39	0,14	7	0,36	0,14	0,17	0,21	1,90	0,14	7	0,04	0,16	0,06	0,04	2,00	0,09	5	0,04	1,40	0,07	0,51	1,40	0,05
13	4	0,15	0,27	0,13	0,40	0,40	0,14	8	0,34	0,18	0,20	0,21	1,60	0,16	6	0,05	0,19	0,21	0,04	2,40	0,11	4	0,04	1,40	0,21	0,35	1,90	0,10
14	2	0,11	0,36	0,11	0,36	0,42	0,07	6	0,31	0,20	0,06	0,20	1,60	0,09	5	0,04	0,16	0,05	0,04	2,20	0,07	4	0,04	1,50	0,09	0,56	2,10	0,10
15	4	0,19	0,29	0,12	0,50	0,53	0,15	7	0,33	0,20	0,13	0,20	0,97	0,08	8	0,04	0,17	0,16	0,04	2,40	0,10	5	0,04	1,30	0,14	0,58	1,50	0,13
16	6	0,14	0,41	0,14	0,36	0,35	0,12	5	0,30	0,11	0,10	0,21	1,60	0,09	6	0,04	0,10	0,17	0,04	2,40	0,04	4	0,05	0,85	0,11	0,43	1,90	0,18
17	4	0,20	0,34	0,07	0,34	0,39	0,13	5	0,33	0,13	0,14	0,20	0,75	0,09	4	0,04	0,12	0,23	0,04	1,70	0,10	4	0,04	0,88	0,14	0,43	1,20	0,07
18	4	0,13	0,39	0,19	0,36	0,38	0,10	6	0,30	0,21	0,02	0,19	1,40	0,12	6	0,05	0,16	0,19	0,04	2,20	0,09	4	0,04	0,67	0,19	0,44	1,70	0,09
19	4	0,09	0,31	0,14	0,46	0,36	0,04	6	0,38	0,20	0,21	0,22	1,70	0,08	8	0,04	0,10	-0,01	0,04	1,70	0,15	4	0,04	1,10	0,09	0,56	2,10	0,16
20	4	0,14	0,38	0,10	0,54	0,45	0,19	6	0,35	0,12	0,04	0,20	1,30	0,09	6	0,04	0,15	0,04	0,04	2,30	0,14	5	0,04	0,75	0,05	0,53	1,30	0,01
21	4	0,10	0,39	0,09	0,58	0,41	0,06	7	0,31	0,08	0,14	0,21	1,70	0,08	5	0,04	0,20	0,01	0,04	2,40	0,08	3	0,04	1,30	0,10	0,55	1,30	0,08
22	4	0,16	0,41	0,10	0,54	0,36	0,06	6	0,35	0,12	0,10	0,22	1,20	0,10	6	0,04	0,19	0,19	0,04	2,40	0,07	3	0,04	1,30	0,10	0,48	1,20	0,17
23	4	0,14	0,30	0,12	0,45	0,36	0,03	7	0,30	0,17	0,12	0,22	1,80	0,06	6	0,03	0,21	0,21	0,04	2,00	0,07	3	0,05	1,20	0,13	0,57	1,50	0,16
24	4	0,24	0,35	0,06	0,41	0,30	0,09	8	0,20	0,15	0,12	0,22	1,40	0,08	6	0,04	0,14	0,16	0,04	2,70	0,16	5	0,04	1,50	0,14	0,32	1,60	0,08
25	4	0,09	0,29	0,08	0,27	0,33	0,09	6	0,29	0,04	0,16	0,21	1,60	0,06	7	0,04	0,11	0,22	0,04	2,20	0,15	4	0,04	1,20	0,13	0,18	2,00	-0,01
26	5	0,20	0,28	0,10	0,36	0,43	0,14	6	0,40	0,19	0,19	0,21	1,10	0,11	6	0,04	0,14	0,16	0,04	1,80	0,05	5	0,04	1,10	0,10	0,55	1,70	0,08
27	2	0,11	0,32	0,15	0,52	0,44	0,17	7	0,28	0,14	0,13	0,22	1,80	0,07	6	0,04	0,23	0,06	0,04	2,10	0,07	4	0,04	1,10	0,11	0,52	1,60	0,11
28	3	0,17	0,36	0,15	0,25	0,38	0,08	6	0,25	0,12	0,22	0,22	1,30	0,10	5	0,04	0,18	0,06	0,04	1,90	0,11	3	0,04	1,20	0,13	0,42	2,40	0,10
29	4	0,20	0,34	0,04	0,35	0,38	0,07	7	0,28	0,08	0,05	0,23	1,50	0,15	7	0,05	0,08	0,15	0,04	1,80	0,11	4	0,05	1,10	0,11	0,50	1,20	0,11
30	4	0,08	0,27	0,16	0,40	0,34	0,12	5	0,37	0,16	0,19	0,22	1,50	0,12	9	0,05	0,17	0,10	0,04	2,90	0,12	4	0,04	0,62	0,06	0,49	1,30	0,10

Pat no	time point 36							time point 39							time point 42							time point 45						
	score	E1	E2	E3	RF	ANA	ACPA	score	E1	E2	E3	RF	ANA	ACPA	score	E1	E2	E3	RF	ANA	ACPA	score	E1	E2	E3	RF	ANA	ACPA
1	4	0,44	0,28	0,16	0,71	0,34	0,25	3	0,70	0,32	0,11	0,04	0,30	0,10	1	0,04	0,46	2,90	0,02	0,46	0,28	3	0,05	0,29	0,17	0,05	0,29	0,10
2	4	0,32	0,26	0,06	0,70	0,31	0,26	5	0,63	0,25	0,08	0,03	0,29	0,08	2	0,04	0,35	3,00	0,04	0,47	0,25	1	0,04	0,22	0,10	0,03	0,36	0,08
3	3	0,39	0,26	0,12	0,83	0,33	0,33	3	0,64	0,23	0,15	0,04	0,24	0,13	3	0,04	0,60	3,60	0,05	0,49	0,31	3	0,04	0,33	0,14	0,04	0,30	0,14
4	3	0,43	0,31	0,19	0,59	0,33	0,44	4	0,72	0,35	0,12	0,03	0,29	0,18	2	0,04	0,56	3,00	0,03	0,48	0,23	1	0,04	0,25	0,11	0,06	0,23	0,04
5	3	0,43	0,36	0,08	0,83	0,19	0,25	5	0,72	0,21	0,09	0,03	0,35	0,13	2	0,04	0,55	2,30	0,04	0,48	0,18	3	0,04	0,25	0,09	0,05	0,35	0,09
6	5	0,34	0,32	0,12	0,54	0,21	0,41	7	0,64	0,20	0,18	0,05	0,30	0,11	2	0,04	0,61	3,60	0,04	0,53	0,32	2	0,04	0,22	0,12	0,06	0,23	0,07
7	4	0,50	0,27	0,08	0,86	0,30	0,28	3	0,65	0,26	0,16	0,03	0,27	0,13	0	0,04	0,51	3,60	0,06	0,50	0,21	2	0,04	0,24	0,07	0,05	0,39	0,18
8	3	0,36	0,23	0,05	0,81	0,36	0,36	2	0,61	0,25	0,05	0,03	0,30	0,18	1	0,04	0,55	3,30	0,04	0,42	0,29	2	0,04	0,29	0,13	0,04	0,27	0,13
9	3	0,39	0,23	0,15	0,59	0,24	0,34	4	0,72	0,33	0,12	0,04	0,37	0,08	3	0,04	0,64	2,50	0,04	0,41	0,15	2	0,03	0,31	0,13	0,05	0,38	0,08
10	3	0,42	0,27	0,12	0,66	0,25	0,30	3	0,66	0,33	0,14	0,05	0,28	0,15	3	0,04	0,72	3,70	0,05	0,62	0,27	3	0,04	0,32	0,12	0,03	0,32	0,10
11	5	0,54	0,24	0,12	0,84	0,36	0,32	5	0,66	0,34	0,08	0,04	0,23	0,16	2	0,04	0,14	3,30	0,04	0,51	0,22	2	0,04	0,28	0,09	0,05	0,36	0,15
12	3	0,42	0,29	0,11	0,74	0,26	0,42	3	0,65	0,23	0,18	0,03	0,33	0,10	2	0,04	0,44	3,80	0,03	0,41	0,31	1	0,04	0,30	0,10	0,05	0,38	0,14
13	3	0,38	0,27	0,09	0,79	0,29	0,25	5	0,58	0,27	0,11	0,03	0,28	0,02	3	0,04	0,48	3,10	0,06	0,37	0,20	1	0,04	0,27	0,10	0,02	0,36	0,03
14	4	0,46	0,33	0,07	0,77	0,30	0,51	4	0,59	0,33	0,17	0,06	0,32	0,08	1	0,04	0,27	1,90	0,04	0,55	0,12	0	0,04	0,23	0,11	0,05	0,34	0,10
15	5	0,46	0,24	0,14	0,72	0,38	0,24	2	0,66	0,23	0,12	0,06	0,34	0,11	2	0,04	0,33	3,00	0,04	0,46	0,19	2	0,05	0,33	0,05	0,04	0,31	0,09
16	3	0,44	0,29	0,17	0,68	0,30	0,29	4	0,67	0,28	0,08	0,04	0,26	0,16	2	0,04	0,48	2,60	0,06	0,55	0,21	3	0,04	0,27	0,11	0,03	0,24	0,11
17	4	0,50	0,28	0,13	0,60	0,29	0,22	5	0,60	0,23	0,09	0,04	0,38	0,12	2	0,04	0,36	2,50	0,04	0,50	0,21	2	0,04	0,32	0,10	0,05	0,27	0,05
18	5	0,53	0,32	0,11	0,49	0,30	0,26	5	0,64	0,28	0,11	0,04	0,23	0,06	1	0,04	0,61	1,50	0,03	0,55	0,14	3	0,05	0,27	0,11	0,04	0,31	0,07
19	6	0,46	0,32	0,06	0,69	0,28	0,40	5	0,72	0,27	0,20	0,04	0,28	0,20	3	0,04	0,23	0,70	0,02	0,51	0,23	4	0,04	0,30	0,14	0,02	0,26	0,17
20	3	0,50	0,29	0,06	0,71	0,30	0,31	5	0,68	0,34	0,10	0,03	0,36	0,09	1	0,04	0,11	4,00	0,05	0,54	0,28	1	0,04	0,27	0,11	0,03	0,27	0,03
21	4	0,43	0,30	0,17	0,77	0,28	0,24	3	0,73	0,34	0,11	0,04	0,32	0,09	1	0,04	0,29	2,90	0,05	0,49	0,23	1	0,04	0,30	0,09	0,05	0,22	0,08
22	5	0,41	0,30	0,12	0,48	0,30	0,37	5	0,67	0,25	0,12	0,05	0,26	0,12	2	0,04	0,87	3,20	0,03	0,54	0,17	3	0,05	0,28	0,07	0,04	0,33	0,10
23	4	0,42	0,28	0,10	0,85	0,26	0,27	3	0,76	0,27	0,07	0,05	0,31	0,09	2	0,04	0,22	3,60	0,04	0,51	0,21	2	0,04	0,29	0,17	0,04	0,30	0,08
24	3	0,38	0,27	0,12	0,76	0,33	0,24	3	0,63	0,23	0,02	0,03	0,34	0,06	3	0,04	0,23	3,10	0,04	0,55	0,26	2	0,04	0,32	0,14	0,03	0,31	0,14
25	4	0,42	0,28	0,04	0,86	0,27	0,27	4	0,63	0,18	0,14	0,04	0,34	0,10	2	0,04	0,91	2,40	0,03	0,49	0,26	3	0,04	0,29	0,09	0,02	0,26	0,13
26	5	0,40	0,40	0,16	0,76	0,34	0,34	5	0,65	0,27	0,04	0,05	0,26	0,08	2	0,04	0,93	2,10	0,05	0,51	0,24	0	0,04	0,26	0,14	0,03	0,26	0,07
27	6	0,39	0,24	0,10	0,82	0,33	0,33	3	0,63	0,24	0,09	0,05	0,32	0,06	0	0,04	0,28	2,50	0,04	0,44	0,28	1	0,04	0,28	0,14	0,04	0,29	0,06

28	4	0,48	0,30	0,10	0,71	0,30	0,26	3	0,69	0,22	0,09	0,03	0,38	0,12	2	0,04	0,37	2,50	0,04	0,49	0,26	3	0,04	0,24	0,13	0,03	0,30	0,10
29	6	0,48	0,31	0,09	0,61	0,24	0,18	5	0,65	0,22	0,10	0,04	0,31	0,09	0	0,04	0,42	3,60	0,04	0,49	0,27	2	0,05	0,29	0,10	0,04	0,27	0,07
30	3	0,42	0,32	0,10	0,96	0,38	0,27	2	0,57	0,25	0,16	0,03	0,20	0,12	2	0,04	0,69	4,50	0,04	0,53	0,17	1	0,04	0,23	0,13	0,05	0,19	0,11

Pat no	time point 48							time point 51							time point 54							time point 57						
	score	E1	E2	E3	RF	ANA	ACPA	score	E1	E2	E3	RF	ANA	ACPA	score	E1	E2	E3	RF	ANA	ACPA	score	E1	E2	E3	RF	ANA	ACPA
1	2	0,04	0,13	0,38	0,05	1,40	0,41	4	0,09	0,12	0,27	0,06	2,20	0,18	4	0,05	0,34	0,41	0,06	2,40	0,31	5	0,07	0,31	0,21	0,41	2,30	0,06
2	0	0,04	0,07	0,21	0,04	2,10	0,30	5	0,17	0,14	0,32	0,05	1,80	0,17	5	0,03	0,32	0,24	0,05	2,20	0,13	8	0,10	0,26	0,32	0,32	2,10	0,11
3	2	0,04	0,09	0,29	0,03	1,80	0,33	4	0,06	0,12	0,17	0,06	1,00	0,18	5	0,08	0,31	0,31	0,07	2,70	0,22	6	0,06	0,30	0,28	0,35	1,80	0,17
4	2	0,04	0,07	0,33	0,06	1,60	0,43	4	0,11	0,11	0,13	0,07	1,00	0,29	5	0,10	0,39	0,23	0,05	1,70	0,24	8	0,07	0,27	0,35	0,37	2,30	0,10
5	2	0,04	0,13	0,28	0,04	1,80	0,29	5	0,09	0,14	0,24	0,06	1,50	0,13	7	0,07	0,32	0,34	0,07	1,90	0,38	4	0,07	0,30	0,35	0,29	1,10	0,08
6	4	0,04	0,10	0,31	0,05	2,20	0,30	3	0,11	0,12	0,31	0,06	1,10	0,23	7	0,05	0,33	0,35	0,05	2,50	0,31	6	0,08	0,36	0,36	0,37	1,30	0,08
7	2	0,04	0,08	0,18	0,04	2,60	0,37	3	0,11	0,14	0,18	0,06	1,30	0,15	5	0,08	0,31	0,34	0,06	2,00	0,20	6	0,05	0,34	0,29	0,33	1,40	0,13
8	2	0,04	0,12	0,31	0,02	1,20	0,36	3	0,05	0,15	0,21	0,06	1,50	0,22	6	0,07	0,31	0,30	0,05	2,20	0,29	5	0,10	0,32	0,35	0,30	3,00	0,07
9	2	0,04	0,08	0,44	0,05	1,50	0,18	5	0,10	0,14	0,27	0,06	1,90	0,18	6	0,05	0,24	0,37	0,05	1,80	0,25	6	0,06	0,25	0,32	0,30	1,70	0,08
10	1	0,05	0,07	0,25	0,04	1,10	0,31	4	0,15	0,10	0,30	0,06	1,50	0,28	5	0,04	0,33	0,35	0,06	1,20	0,15	6	0,03	0,35	0,38	0,38	2,10	0,12
11	1	0,04	0,10	0,23	0,05	1,40	0,39	5	0,03	0,14	0,28	0,06	0,90	0,27	7	0,05	0,32	0,28	0,07	1,30	0,42	6	0,06	0,27	0,21	0,46	2,30	0,13
12	3	0,04	0,10	0,42	0,03	2,30	0,28	3	0,07	0,11	0,34	0,06	1,40	0,21	7	0,08	0,30	0,34	0,06	3,10	0,34	6	0,06	0,32	0,18	0,56	2,60	0,12
13	3	0,04	0,10	0,32	0,05	2,00	0,41	5	0,12	0,10	0,25	0,06	1,70	0,18	9	0,10	0,34	0,22	0,06	2,70	0,32	7	0,08	0,34	0,27	0,48	2,60	0,08
14	1	0,04	0,12	0,41	0,03	2,20	0,37	4	0,14	0,14	0,34	0,06	1,30	0,30	7	0,08	0,27	0,36	0,06	2,20	0,30	6	0,07	0,24	0,22	0,40	2,70	0,09
15	3	0,04	0,12	0,27	0,04	2,00	0,33	3	0,10	0,18	0,26	0,05	1,10	0,10	7	0,08	0,25	0,33	0,06	2,80	0,42	7	0,04	0,28	0,38	0,49	2,00	0,18
16	2	0,04	0,09	0,27	0,05	1,50	0,34	3	0,13	0,11	0,24	0,05	0,99	0,32	5	0,08	0,34	0,36	0,07	2,40	0,31	4	0,08	0,28	0,35	0,50	1,20	0,06
17	4	0,05	0,09	0,30	0,04	0,79	0,44	5	0,17	0,12	0,35	0,05	1,30	0,21	4	0,04	0,32	0,22	0,06	1,20	0,26	6	0,08	0,35	0,30	0,26	2,50	0,22
18	2	0,03	0,05	0,37	0,05	1,60	0,29	4	0,06	0,10	0,39	0,06	1,50	0,30	7	0,06	0,21	0,27	0,05	2,40	0,47	7	0,07	0,26	0,33	0,47	2,20	0,12
19	3	0,04	0,05	0,26	0,04	1,30	0,41	4	0,17	0,14	0,26	0,06	1,50	0,19	5	0,08	0,27	0,40	0,07	1,50	0,24	5	0,09	0,35	0,46	0,34	2,80	0,12
20	3	0,04	0,11	0,37	0,03	1,60	0,27	4	0,10	0,15	0,25	0,06	2,10	0,13	5	0,09	0,32	0,31	0,06	2,70	0,24	5	0,10	0,27	0,26	0,30	2,90	0,08
21	2	0,04	0,11	0,25	0,05	1,60	0,37	5	0,03	0,11	0,23	0,06	0,55	0,22	5	0,08	0,23	0,27	0,06	2,60	0,47	5	0,08	0,32	0,32	0,30	1,40	0,06
22	2	0,04	0,10	0,32	0,04	1,80	0,30	4	0,05	0,12	0,33	0,06	0,99	0,30	6	0,08	0,40	0,28	0,06	2,00	0,41	8	0,07	0,33	0,35	0,35	1,50	0,09
23	0	0,04	0,08	0,33	0,02	2,30	0,42	5	0,19	0,16	0,31	0,06	1,30	0,25	7	0,08	0,26	0,31	0,07	1,20	0,29	5	0,07	0,35	0,24	0,38	2,20	0,14
24	1	0,04	0,10	0,45	0,04	2,10	0,36	2	0,14	0,14	0,21	0,04	1,20	0,17	6	0,09	0,31	0,39	0,05	3,20	0,51	7	0,10	0,33	0,33	0,20	1,70	0,17
25	2	0,04	0,09	0,33	0,05	1,90	0,41	5	0,12	0,13	0,43	0,06	1,20	0,22	5	0,09	0,35	0,25	0,06	2,90	0,37	6	0,07	0,31	0,23	0,39	2,30	0,01
26	2	0,04	0,08	0,44	0,02	2,50	0,38	5	0,19	0,12	0,48	0,06	1,70	0,13	7	0,05	0,27	0,39	0,07	2,60	0,30	4	0,06	0,33	0,46	0,40	1,80	0,08
27	2	0,04	0,09	0,25	0,05	1,30	0,43	3	0,04	0,11	0,29	0,07	1,30	0,12	5	0,04	0,21	0,24	0,07	2,00	0,52	7	0,10	0,30	0,26	0,38	1,90	0,13
28	2	0,04	0,10	0,26	0,05	1,60	0,37	4	0,21	0,14	0,40	0,06	2,60	0,28	5	0,07	0,29	0,38	0,06	2,70	0,12	6	0,08	0,36	0,23	0,39	2,10	0,11
29	2	0,03	0,07	0,31	0,04	1,80	0,30	6	0,16	0,15	0,22	0,05	1,00	0,29	6	0,07	0,28	0,36	0,05	2,10	0,30	5	0,06	0,28	0,29	0,27	2,00	0,16
30	1	0,04	0,09	0,31	0,03	2,00	0,33	4	0,12	0,13	0,23	0,05	0,81	0,19	6	0,06	0,34	0,16	0,05	2,80	0,31	6	0,08	0,31	0,31	0,35	2,60	0,08

Pat no	time point 60						
	score	E1	E2	E3	RF	ANA	ACPA
1	5	0,32	0,28	0,04	0,16	1,70	0,37
2	4	0,20	0,26	0,05	0,14	1,40	0,30
3	3	0,33	0,34	0,04	0,19	1,10	0,24
4	5	0,39	0,34	0,03	0,21	0,56	0,31
5	3	0,34	0,27	0,04	0,27	1,00	0,28
6	5	0,20	0,29	0,04	0,18	0,97	0,37
7	2	0,34	0,26	0,04	0,32	1,20	0,28
8	4	0,25	0,38	0,05	0,44	1,30	0,30
9	5	0,36	0,28	0,04	0,33	1,30	0,28
10	5	0,28	0,23	0,05	0,32	0,67	0,23
11	4	0,37	0,26	0,03	0,17	1,50	0,27
12	4	0,38	0,25	0,04	0,31	0,96	0,39
13	3	0,30	0,29	0,04	0,44	0,50	0,42
14	4	0,33	0,26	0,03	0,23	1,30	0,19
15	3	0,28	0,25	0,03	0,10	1,10	0,33
16	5	0,27	0,31	0,04	0,19	0,61	0,28
17	3	0,36	0,23	0,04	0,16	0,89	0,35
18	4	0,28	0,28	0,04	0,24	0,84	0,33
19	4	0,36	0,34	0,03	0,15	0,99	0,25
20	4	0,37	0,36	0,02	0,31	1,60	0,31
21	4	0,38	0,25	0,04	0,18	1,80	0,22
22	5	0,17	0,30	0,05	0,24	1,70	0,27
23	5	0,24	0,30	0,05	0,11	1,50	0,21
24	6	0,34	0,25	0,04	0,19	1,40	0,42
25	5	0,29	0,31	0,04	0,45	2,70	0,30
26	4	0,39	0,32	0,04	0,17	1,50	0,20
27	4	0,41	0,24	0,04	0,04	1,60	0,13
28	3	0,33	0,32	0,05	0,32	1,70	0,29
29	4	0,34	0,28	0,04	0,27	1,10	0,22
30	4	0,35	0,32	0,04	0,38	1,40	0,32

ELISA has been carried out as described in Methods. Randomly selected 5% of samples have been tested in triplicate using the same plate. According to chi2 statistical test, 5% have been a sufficient sampling to represent the entire cohort (30 out of 600 total observations for each test; chi-square statistic is 33.2908. The p-value is < 0.00001). CV values were within the range given in Methods section. Below, individual data points for all subjects are given.
