# Supplementary material for: Antibodies to synthetic citrullinated peptide epitope correlate with disease activity and flares in rheumatoid arthritis
Source: PLoS One. 2020 Apr 23;15(4):e0232010. doi: 10.1371/journal.pone.0232010 (PMC7179858; doi:10.1371/journal.pone.0232010)
Supplement: S8 Appendix — S12 Table. E2 ELISA results (A450 values), for individual samples: healthy controls (HC), JIA, SSc, SLE and RA. S14 Table. Individual data points for serum dilution study, 20 RA patients. S1 Fig. Results of ROC for E2 (A) and CCP2 (B). (PDF) [file pone.0232010.s008.pdf]

S8 Appendix. Individual values for ELISA of healthy and diseased controls; ROC analysis

ELISA has been carried out as described in Methods. Randomly selected 5% of samples have been tested in triplicate using the same plate. According to chi2 statistical test, 5% have been a sufficient sampling to represent the entire cohort (31 out of 624 total observations; chi-square statistic is 8.8181. The p-value is 0.002983). CV values were within the range given in Methods section. Below, individual data points for all subjects are given.

S12 Table. E2 ELISA results (A450 values), for individual samples: healthy controls (HC), JIA, SSc, SLE and RA.

HC	JIA	SSc	SLE	RA
0.32	0.39	0.13	0.36	0.21
0.30	0.37	0.07	0.34	1.00
0.31	0.38	0.87	0.35	0.90
0.14	0.17	0.67	0.16	1.00
0.44	0.54	0.10	0.49	0.81
0.26	0.32	0.08	0.29	1.11
0.38	0.47	0.07	0.43	0.45
0.73	0.90	0.13	0.82	1.10
0.27	0.33	0.33	0.30	0.60
0.45	0.55	0.32	0.50	0.89
0.37	0.46	0.04	0.41	0.55
0.23	0.28	0.33	0.26	1.10
0.58	0.71	0.32	0.65	0.76
0.37	0.46	0.05	0.41	1.10
0.10	0.43	0.12	0.11	0.54
0.10	0.12	0.23	0.11	0.43
0.21	0.26	0.21	0.24	0.55
0.84	1.03	0.13	0.94	1.21
0.11	0.14	0.07	0.12	1.40
0.66	0.81	0.19	0.74	1.20
0.22	0.27	0.68	0.25	0.34
0.76	0.93	0.10	0.85	0.65
0.44	0.54	0.08	0.49	0.11
0.12	0.15	0.07	0.13	0.17
0.23	0.28	0.13	0.26	1.34
0.19	0.23	0.33	0.21	0.40
0.55	0.68	0.32	0.62	0.43
0.11	0.14	0.04	0.12	0.21
0.55	0.68	0.08	0.10	0.22

0.82	1.01	0.11	0.83	0.44
0.20	0.24	0.27	0.20	0.55
0.28	0.35	0.13	0.98	
0.24	0.29	0.37	0.20	
1.20	1.48	0.23	0.40	
0.21	0.26	0.24	0.50	
0.17	0.21	0.37	0.24	
0.18	0.22	0.14	1.00	
0.26	0.31	0.26	2.30	
0.10	0.13	0.06	0.09	
0.19	0.43	0.34	1.32	
0.18	0.54	0.50	0.51	
0.16	0.67	0.07	0.54	
0.21	0.66	0.05	0.34	
0.21	0.89	0.07	0.51	
0.21	0.43	0.11	1.40	
0.25	0.40	0.08	0.30	
0.20	0.58	0.09	0.10	
0.26	0.32	0.25	0.57	
0.27	0.33	0.21	0.40	
0.45	0.55	0.24	2.29	
0.26	0.33	0.11	1.70	
0.23	0.28	0.08	1.10	
0.17	0.21	0.11	2.10	
0.18	0.22	0.27	1.70	
0.10		0.14	1.10	
0.27		0.22	1.60	
0.19		0.08	0.20	
0.12		0.11	0.91	
0.23		0.27	0.10	
0.80		0.13	0.11	
		0.37	0.32	
		0.23	0.30	
		0.24	1.32	
		0.37	0.21	
		0.14	0.30	
		0.26	1.36	
		0.06	0.05	
		0.34	0.20	
		0.50	0.49	
		0.07	0.05	
		0.05	1.60	

0.07	0.11
0.11	0.21
0.08	0.38
0.09	0.60
0.25	0.08
0.21	1.40
0.24	0.20
0.11	0.10
0.08	0.27
0.11	1.30
0.27	0.80
0.14	0.30
0.22	0.40
0.11	0.10
0.06	0.11
0.34	0.22
0.22	0.31
0.11	0.17
0.23	0.10
	0.22
	0.08
	0.63
	0.16
	0.99
	0.49
	0.52
	0.99
	0.16
	0.60
	0.07
	0.38
	0.40
	0.21
	0.12
	0.69
	0.08
	0.22
	0.03
	0.58
	0.41
	0.52
	0.11
	0.09
	0.08

0.63
0.16
0.32
0.34
0.32
0.05
0.12
0.23
0.22
0.14
0.07
0.19
0.70
0.10
0.08
0.22
0.10
0.86
0.12
0.23
0.19
0.25
0.11
0.07
1.80
0.08
0.11
0.27
0.13
0.37
0.23
0.24
0.37
0.14
0.26
0.06
0.34
0.50
0.07
0.05
0.07
0.11
0.08
0.09
0.25

0.21
0.24
0.11
0.08
0.11
0.27
0.14
0.22
0.08
0.32
0.12
0.36
0.10
0.09
0.08
1.47
0.08
0.11
0.27
0.13
0.37
0.23
0.24
0.37
0.10
0.07
0.12
0.25
0.20
0.26
0.11
0.06
1.20
0.11
0.12
0.31
0.36
0.35
0.04
0.12
0.25
0.23
0.14
0.07

0.20
0.77
0.10
0.08
0.23
0.10
0.07
0.12
0.25
0.20
0.26
0.11
0.06
0.02
1.22
0.11
0.23
0.33
0.18
0.10
0.43
0.41
0.42
0.19
0.60
0.35
0.51
0.99
0.37
0.61
0.50
0.31
0.79
0.50
0.14
0.14
0.28
1.14
0.15
0.89
0.30
0.03
0.60
0.16

0.31
0.26
0.21
0.15
0.12
0.04
0.24
1.19
0.24
0.48
0.61
0.29
0.42
0.09
0.11
1.60
0.62
0.65
0.41
0.62
1.69
0.36
0.12
0.69
0.48
0.77
0.06
0.33
0.54
0.06
1.33
1.94
0.24
1.10
0.12
0.13
0.59
0.36
0.61
0.25
0.36
0.86
0.06
0.24

0.59
0.06
0.14
0.13
0.25
0.46
0.73
0.10
0.21
0.66
0.54
0.33
0.12
0.47
0.17
0.19
0.23
0.13
0.27
0.38
0.21
0.12
0.27
0.10
0.76
0.19
1.20
0.59
0.63
0.20
0.19
0.73
0.08
0.06
0.31
0.25
0.15
0.25
0.10
0.27
0.04
0.70
0.50
0.63

0.13
0.11
0.10
0.76
0.19
0.39
0.41
0.39
0.06
0.15
0.28
0.27
0.17
0.08
0.23
0.85
0.12
0.10
0.27
0.12
2.25
0.15
0.28
0.23
0.30
0.13
0.08
0.18
0.10
0.13
0.33
0.16
0.45
0.28
0.29
0.45
0.17
0.31
0.07
0.41
0.61
0.08
0.06
0.08
0.13

0.10
0.11
0.30
0.25
0.29
0.13
0.10
0.13
0.33
0.17
0.27
0.10
0.39
0.23
0.14
0.54
0.40
0.10
1.78
0.10
0.13
0.33
0.16
0.45
0.28
0.29
0.45
0.12
0.02
0.15
0.30
0.24
0.31
0.13
0.07
1.45
0.13
0.15
0.38
0.44
0.42
0.05
0.15
0.30

0.28

S14 Table. Individual data points (A450, ELISA) for serum dilution study, 20 RA patients.

							Replicate measurements					
pat no	dilution factor 1000 CCP2	anti-E2	dilution factor 5000 CCP2	anti-E2	dilution factor 10000 CCP2	anti-E2	dilution factor 1000 CCP2	anti-E2	dilution factor 5000 CCP2	anti-E2	dilution factor 10000 CCP2	anti-E2
1	2.70	4.50	0.46	1.60	0.27	0.49	2.40	4.60	0.55	1.60	0.22	0.54
2	2.40	5.20	0.48	1.70	0.27	1.10	2.40	5.10	0.48	1.72	0.20	1.12
3	2.70	4.40	0.40	1.80	0.21	0.90	2.70	4.30	0.42	1.70	0.22	0.94
4	2.10	4.20	0.47	1.80	0.23	0.96	1.80	4.40	0.41	1.75	0.17	0.90
5	2.10	4.10	0.46	1.50	0.24	0.76	2.30	3.60	0.44	1.40	0.20	0.70
6	1.80	4.50	0.45	1.00	0.21	0.40	1.80	4.40	0.45	0.98	0.24	0.33
7	2.30	4.40	0.43	1.80	0.21	0.28	2.50	4.90	0.43	1.70	0.17	0.20
8	1.80	4.50	0.38	1.10	0.25	0.69	1.80	4.60	0.39	1.00	0.43	0.60
9	2.30	4.80	0.40	2.00	0.26	0.91	2.32	4.80	0.40	2.12	0.26	0.81
10	1.50	4.60	0.47	1.40	0.23	0.79	1.52	4.50	0.40	1.40	0.20	0.65
11	2.50	4.30	0.43	1.50	0.23	1.50	2.50	4.30	0.40	1.50	0.21	1.44
12	2.00	3.90	0.48	1.70	0.25	0.93	2.00	4.10	0.44	1.71	0.17	0.80
13	1.90	4.60	0.47	1.50	0.19	0.52	1.95	4.60	0.49	1.55	0.19	0.40
14	1.60	4.20	0.52	1.60	0.21	1.10	1.70	4.20	0.52	1.56	0.18	0.78
15	3.00	3.90	0.49	1.70	0.21	0.47	3.00	3.90	0.49	1.89	0.22	0.41
16	2.40	4.20	0.42	2.20	0.25	1.20	2.50	4.10	0.37	2.11	0.34	1.01
17	2.90	4.10	0.40	1.40	0.27	1.20	2.92	4.40	0.36	1.45	0.22	1.43
18	2.10	4.60	0.43	1.20	0.19	1.40	1.90	4.60	0.40	1.20	0.19	1.22
19	2.30	4.50	0.49	1.50	0.17	1.00	2.20	4.70	0.49	1.45	0.17	1.00
20	2.40	3.80	0.43	1.40	0.22	0.45	2.30	3.90	0.40	1.44	0.20	0.44

							Replicate measurements					
pat no	dilution factor 20000 CCP2	anti-E2	dilution factor 80000 CCP2	anti-E2	dilution factor 100000 CCP2	anti-E2	dilution factor 20000 CCP2	anti-E2	dilution factor 80000 CCP2	anti-E2	dilution factor 100000 CCP2	anti-E2
1	0.04	0.28	0.0010	0.13	0.0004	0.11	0.04	0.29	0.0010	0.13	0.0004	0.11
2	0.07	0.36	0.0010	0.18	0.0008	0.11	0.07	0.37	0.0010	0.19	0.0009	0.11
3	0.06	0.33	0.0010	0.13	0.0017	0.14	0.07	0.34	0.0010	0.13	0.0018	0.15
4	0.06	0.35	0.0010	0.18	0.0034	0.13	0.07	0.36	0.0010	0.19	0.0035	0.14
5	0.13	0.60	0.0010	0.18	0.0074	0.18	0.13	0.62	0.0010	0.19	0.0076	0.19
6	0.18	0.32	0.0010	0.10	0.0014	0.12	0.19	0.33	0.0010	0.10	0.0014	0.13
7	0.07	0.45	0.0010	0.18	0.0024	0.07	0.07	0.46	0.0010	0.19	0.0025	0.07
8	0.12	0.33	0.0010	0.15	0.0025	0.14	0.12	0.34	0.0010	0.14	0.0026	0.15
9	0.11	0.40	0.0010	0.15	0.0021	0.10	0.11	0.41	0.0010	0.15	0.0022	0.10
10	0.13	0.63	0.0010	0.14	0.0050	0.16	0.13	0.63	0.0010	0.14	0.0052	0.17
11	0.08	0.44	0.0010	0.19	0.0026	0.08	0.08	0.44	0.0010	0.22	0.0026	0.09
12	0.09	0.29	0.0010	0.17	0.0022	0.14	0.09	0.29	0.0010	0.18	0.0022	0.15
13	0.10	0.24	0.0010	0.14	0.0023	0.16	0.11	0.24	0.0010	0.14	0.0023	0.17
14	0.11	0.36	0.0010	0.14	0.0013	0.12	0.14	0.37	0.0010	0.14	0.0013	0.13
15	0.15	0.34	0.0010	0.15	0.0005	0.15	0.15	0.35	0.0010	0.15	0.0005	0.16
16	0.06	0.42	0.0010	0.08	0.0038	0.11	0.06	0.43	0.0010	0.08	0.0039	0.11
17	0.08	0.35	0.0010	0.11	0.0008	0.08	0.08	0.36	0.0010	0.11	0.0009	0.08
18	0.11	0.54	0.0010	0.13	0.0003	0.13	0.11	0.56	0.0010	0.13	0.0003	0.14
19	0.10	0.41	0.0010	0.20	0.0025	0.10	0.10	0.42	0.0010	0.21	0.0026	0.10
20	0.02	0.38	0.0010	0.17	0.0065	0.10	0.02	0.38	0.0010	0.18	0.0067	0.10

ROC analysis

A)

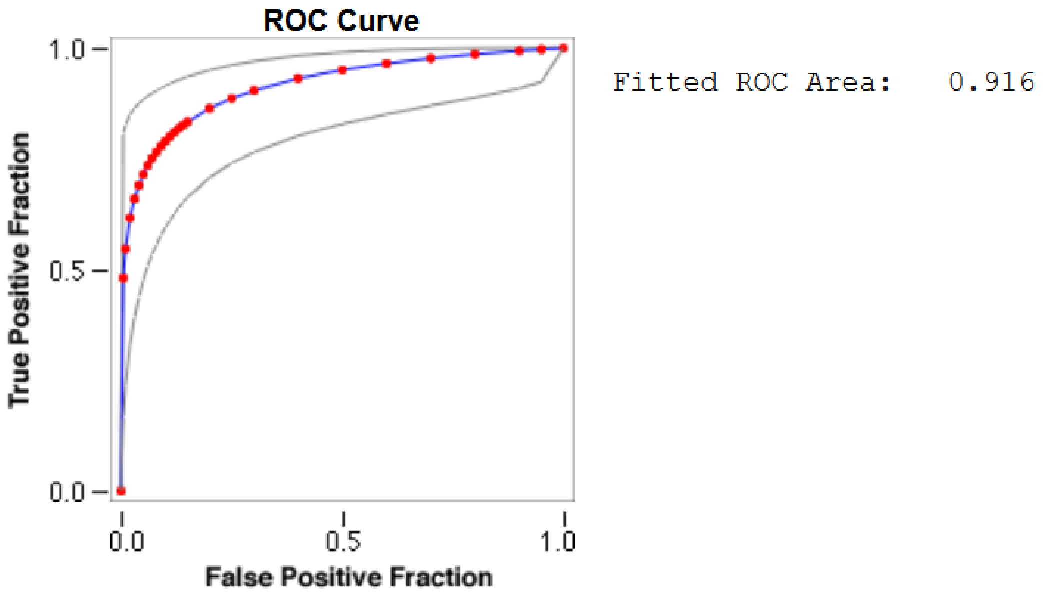

B)

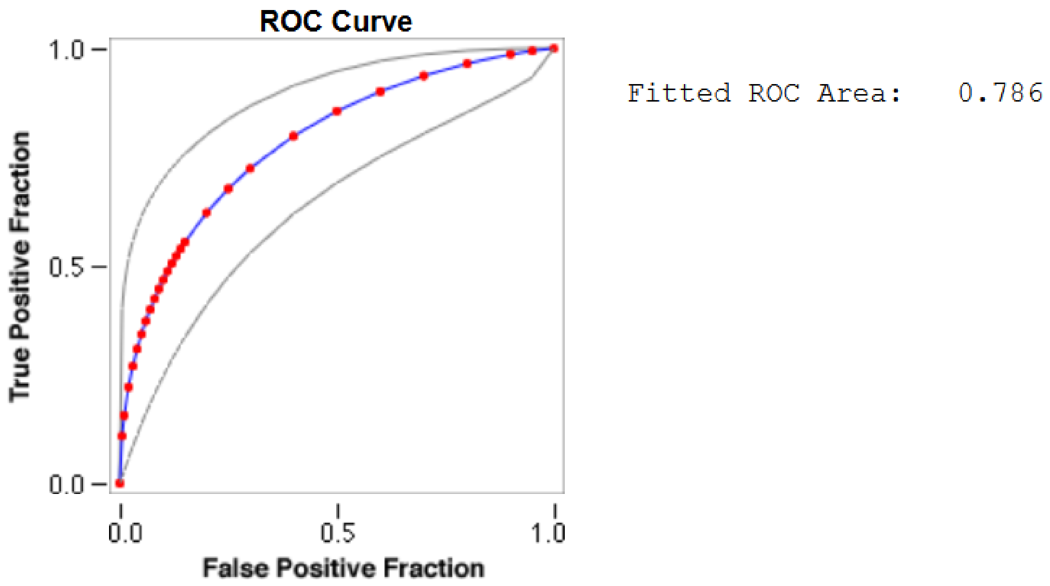

S1 Figure. Results of ROC for E2 (A) and CCP2 (B). Analysis has been conducted using John Hopkins online statistical package.[1]

[1] Web: http://www.rad.jhmi.edu/jeng/javarad/roc/JROCFITi.html
