# Supplementary material for: Antibodies to synthetic citrullinated peptide epitope correlate with disease activity and flares in rheumatoid arthritis
Source: PLoS One. 2020 Apr 23;15(4):e0232010. doi: 10.1371/journal.pone.0232010 (PMC7179858; doi:10.1371/journal.pone.0232010)
Supplement: S9 Appendix — S13. Table. Data for RA individuals applied in joint count correlation study. (PDF) [file pone.0232010.s009.pdf]

S9 Appendix. Joint count correlation study; data for individual patients.

S13. Table. Data for RA individuals applied in joint count correlation study.

					Replicated measurement			
joint count	anti-E1	anti-E2	RF	CCP2	anti-E1	anti-E2	RF	CCP2
2	1.30	1.66	0.12	1.32	1.33	1.71	0.13	1.17
3	1.80	1.66	0.31	1.15	1.84	1.66	0.32	1.21
2	1.10	1.66	0.26	1.10	1.12	1.69	0.27	0.97
4	2.30	1.88	0.29	1.15	2.35	1.94	0.30	1.02
5	3.10	2.76	0.80	1.32	3.16	2.84	0.83	1.17
3	1.80	1.40	0.32	1.17	1.84	1.44	0.33	1.04
2	1.30	1.55	0.22	2.40	1.33	1.60	0.23	2.12
6	2.60	2.22	0.65	1.23	2.65	2.22	0.68	1.09
5	2.50	2.50	1.10	1.30	2.55	2.58	1.15	1.15
4	1.78	1.87	0.45	1.46	1.82	1.93	0.47	1.29
7	4.30	3.27	2.20	1.18	4.39	3.37	2.29	1.04
7	4.40	2.54	2.10	1.32	4.49	2.62	2.19	1.17
4	3.20	2.30	0.46	1.90	3.27	2.37	0.48	1.68
3	2.50	1.66	0.17	1.40	2.55	1.71	0.18	1.24
1	1.23	1.33	0.32	1.43	1.26	1.37	0.33	1.27
0	0.32	1.00	0.09	2.50	0.33	1.03	0.09	2.21
0	0.40	1.01	0.41	2.20	0.41	1.04	0.43	1.95
2	2.20	1.44	0.60	1.23	2.24	1.48	0.63	1.09
3	2.30	1.98	0.70	1.32	2.35	2.04	0.73	1.17
4	2.40	1.88	0.80	1.54	2.45	1.94	0.83	1.36
6	2.30	2.95	1.90	1.98	2.35	3.04	1.98	1.75
4	2.65	1.88	0.15	1.10	2.70	1.94	0.16	0.97
3	2.20	1.98	0.11	0.67	2.24	2.04	0.11	0.59
6	2.10	2.32	0.12	1.65	2.14	2.39	0.13	1.46

7	1.45	3.27	0.31	2.10	1.48	3.27	0.32	1.86
4	1.23	1.88	0.26	2.2	1.26	1.94	0.27	2.21
